# A Comprehensive Survey of Enabling and Emerging Technologies for Social Distancing—Part I: Fundamentals and Enabling Technologies

**DOI:** 10.1109/ACCESS.2020.3018140

**Published:** 2020-08-20

**Authors:** Cong T. Nguyen, Yuris Mulya Saputra, Nguyen Van Huynh, Ngoc-Tan Nguyen, Tran Viet Khoa, Bui Minh Tuan, Diep N. Nguyen, Dinh Thai Hoang, Thang X. Vu, Eryk Dutkiewicz, Symeon Chatzinotas, Björn Ottersten

**Affiliations:** 1 Department of Computer Science and EngineeringHo Chi Minh City University of Technology 117295 Ho Chi MInh City 700000 Vietnam; 2 Department of Computer Science and EngineeringVietnam National University-Ho Chi Minh City 54800 Ho Chi MInh City 700000 Vietnam; 3 School of Electrical and Data EngineeringUniversity of Technology Sydney 1994 Sydney NSW 2007 Australia; 4 Interdisciplinary Centre for Security, Reliability and Trust, University of Luxembourg 81872 4365 Luxembourg City Luxembourg; 5 Department of Electrical Engineering and InformaticsVocational CollegeUniversitas Gadjah Mada 59166 Yogyakarta 55281 Indonesia; 6 VNU University of Engineering and Technology, Vietnam National University Hanoi 711000 Vietnam

**Keywords:** Social distancing, pandemic, COVID-19, wireless, networking, positioning systems, AI, machine learning, data analytics, localization, privacy-preserving, scheduling, incentive mechanism

## Abstract

Social distancing plays a pivotal role in preventing the spread of viral diseases illnesses such as COVID-19. By minimizing the close physical contact among people, we can reduce the chances of catching the virus and spreading it across the community. This two-part paper aims to provide a comprehensive survey on how emerging technologies, e.g., wireless and networking, artificial intelligence (AI) can enable, encourage, and even enforce social distancing practice. In this Part I, we provide a comprehensive background of social distancing including basic concepts, measurements, models, and propose various practical social distancing scenarios. We then discuss enabling wireless technologies which are especially effect- in social distancing, e.g., symptom prediction, detection and monitoring quarantined people, and contact tracing. The companion paper Part II surveys other emerging and related technologies, such as machine learning, computer vision, thermal, ultrasound, etc., and discusses open issues and challenges (e.g., privacy-preserving, scheduling, and incentive mechanisms) in implementing social distancing in practice.

## I. Introduction

COVID-19 has completely changed the world’s view on pandemics with dire consequences to global health and economy. Started in Wuhan, China [2], within only six months (from January to June 2020), 210 countries and territories around the world have reported more than ten million infected people including more than five hundred thousand deaths [3]. Besides the global health crisis, COVID-19 has also been causing massive economic losses (e.g., a possible 25% unemployment rate in the U.S. [4], one million people lost their jobs in Canada during March 2020 [5], 1.4 million jobs lost in Australia [6], and a projected global 3% GDP loss [7]), resulting in a global recession as predicted by many experts [7]–[9]. In such context, there is an urgent need for solutions to contain the disease spread, thereby reducing its negative impacts and buying more time for pharmaceutical solution development.

**FIGURE 1.**
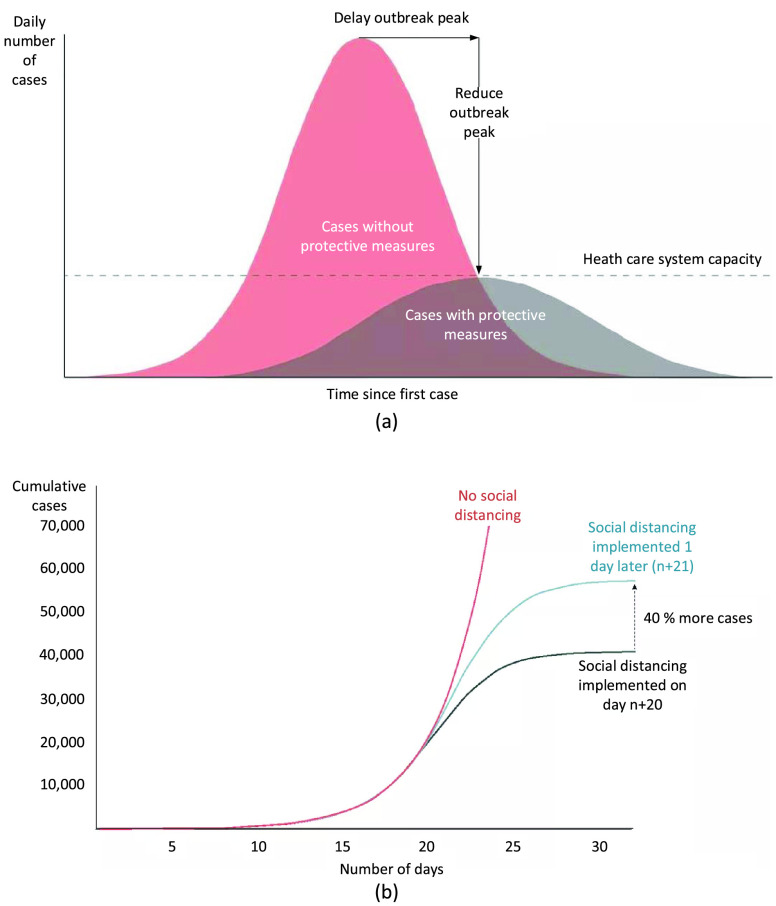
The effects of social distancing on an infected disease outbreak. (a) Social distancing can delay and reduce the outbreak peak. (b) Social distancing can reduce the total number of cases. The earlier social distancing is applied, the stronger its effect will be [12].

In the presence of contagious diseases such as SARS, H1N1, and COVID-19, social distancing is an effective non-pharmaceutical approach to limit the disease transmission [10], [25], [32]. Social distancing refers to measures that minimize the disease spread by reducing the frequency and closeness of human physical contacts, such as closing public places (e.g., schools and workplaces), avoiding mass gatherings, and keeping a sufficient distance amongst people [10], [16]. By reducing the probability that the disease can be transmitted from an infected person to a healthy one, social distancing can significantly reduce the disease’s spread and severity. If implemented properly at the early stages of a pandemic, social distancing measures can play a key role in reducing the infection rate and delay the disease’s peak, thereby reducing the burden on the healthcare systems and lowering death rates [10], [25], [32]. Fig. 1 illustrates the effects of social distancing measures on the daily number of cases [12]. As can be observed in Fig. 1(a), social distancing can reduce the peak number of infected cases [32] to ensure that the number of patients does not exceed the public healthcare capacity. Moreover, social distancing also delays the outbreak peak [32] so that there is more time to implement countermeasures. Furthermore, social distancing can reduce the final number of infected cases [32], and the earlier social distancing is implemented, the stronger the effects will be as illustrated in Fig. 1(b) [12].

During the ongoing COVID-19 pandemic, many governments have implemented various social distancing measures such as travel restrictions, border control, closing public places, and warning their citizens to keep a 1.5–2 meters distance from each other when they have to go outside [13]–[15]. Nevertheless, such aggressive and large-scale measures are not easy to implement, e.g., not all public spaces can be closed, and people still have to go outside for food, healthcare, or essential work. In such context, technologies play a key role in facilitating social distancing measures. For example, wireless positioning systems can effectively help people to keep a safe distance by measuring the distances among people and alerting them when they are too close to each other. Moreover, other technologies such as Artificial Intelligence (AI) technologies can be used to facilitate or even enforce social distancing.

In this two-part paper, we present a comprehensive survey on enabling and emerging technologies for social distancing. The main aims are to provide a comprehensive background on social distancing as well as effective technologies that can be used to facilitate the social distancing practice. In Part I, we first present basic concepts of social distancing together with its measurements, models, effectiveness, and practical scenarios. After that, we review enabling wireless technologies which are especially effective in monitoring and keeping distance amongst people. In Part II [1], we survey other emerging technologies, e.g., AI, thermal, computer vision, ultrasound, and visible light, and discuss open issues and challenges (e.g., privacy-preserving, scheduling, and incentive mechanisms) of implementing technologies for social distancing.

There are several surveys of enabling technologies for the current COVID-19 pandemic, such as [18], [19], with different focuses. Particularly, [18] surveys the application of AI technologies and data-sharing methods for urban health monitoring, and [19] focuses on emerging technologies such as AI, 3D printing, blockchain, etc., and their applications for social distancing. Different from these surveys, our paper focuses on both the newly emerged technologies and the readily available wireless technologies such as Wi-Fi, Bluetooth, Cellular, etc. Moreover, although there are few surveys related to localization and positioning systems using those wireless technologies, e.g., [20]–[23], to the best of our knowledge, this is the first survey in the literature discussing the applications of those technologies for social distancing. It is worth noting that, due to the increasingly complex development of many types of viruses as well as the rapid growth of social interaction and globalization, the concept of social distancing is not as simple as physical distancing. In fact, it also includes many non-pharmaceutical interventions or measures taken to prevent the spread of contagious diseases, such as monitoring, detection, and warning people (as we identify and propose in Table 1). Thanks to the significant development of emerging technologies, e.g., future wireless systems, AI, and data analytics, many new solutions have been introduced recently which can create favorable conditions for practicing social distancing.

**TABLE 1:** Practical Social Distancing Scenarios

	Scenarios	Description	Technologies	References
Keeping Distance	Distance between any two people	Detect and monitor the distance between any two people	Bluetooth, Ultrasound, Thermal, Inertial, Ultra-wideband	[113], [121], [176], [181], [208]
	Distance to/from crowds	Alert when approaching a crowd	AI, Thermal, Inertial, Ultra-wideband, Computer Vision	[122], [180], [186], [208], [216]
Real-time Monitoring	Public place monitoring	Monitor and gauge the number of people inside/at a public place	Wi-Fi, RFID, Zigbee, Cellular, GNSS, Ultrasound, Computer Vision	[129], [130]
	Physical contact monitoring	Monitor physical contacts, e.g., handshakes, hugs, between people	Computer Vision, Thermal	[180], [181]
	Symptom detection and monitoring	Detect and monitor sickness symptoms, e.g., body temperature, coughs	Computer Vision, Thermal, AI	[174]
	Susceptible group detection	Monitor highly susceptible groups	Thermal	[184], [185]
	Detect and monitor quarantined people	Detect and monitor quarantine people (e.g., for complying/violating the isolation/quarantine requirement)	AI, Cellular, GNSS, Ultrasound, Visible Lights, Computer Vision	[175], [218]
	Crowd detection	Detect crowds/gatherings in public places	Wi-Fi, Bluetooth, RFID, Zigbee, AI, Ultra-wideband, Cellular, GNSS, Computer Vision, Visible Light	[55], [75], [111], [121], [150], [159]
	Non-essential travel detection	Using location information to determine if the trip is essential (e.g., medical facilities and gasoline stations) or not (e.g., restaurants and cinemas)	Cellular, GNSS, Thermal	[146], [147], [183]
	Traffic/movement monitoring	Detect the vehicles on the street when isolation measures are in effect	GNSS, Computer Vision, Cellular	[77], [146], [147]
	Lockdown violation detection	Detect violations of public place’s closure or lockdown.	GNSS, Cellular, Thermal	[77], [146], [147], [182]
Information System	Infected movement data	Track the infected people’s movement to notify susceptible people who were at the same places	Cellular, Blockchain, GNSS, Computer Vision	[109], [110], [187]–[190]
	Contact tracing	Trace the contacts that an infected individual made	Bluetooth, Blockchain Ultra-wideband, Thermal, AI	[103], [109], [110], [121], [181], [216]
Incentive	Location/movement sharing encouragement	Encourage people to share their movement data	Bluetooth, Blockchain, incentive mechanism	[103], [106], [109], [110], [223]
	Stay-at-home encouragement	Incentivize people to stay home	Wi-Fi, Cellular, incentive mechanism, optimization	[93], [94], [207], [225], [226]
Scheduling	Workforce scheduling	Limit the number of people at the workplaces	Scheduling	[191]–[194]
	Medical/health appointment scheduling	Schedule medical appointments to reduce the number of patients	Scheduling	[195]–[199]
	Home healthcare scheduling	Optimize home healthcare services to reduce the number of patients at the hospitals	Scheduling	[200]–[204]
	Public place/building access scheduling	Control the number of people inside public buildings	Ultra-wideband, Wi-Fi, RFID, Zigbee	[59], [130], [150], [159]
	Traffic control	Regulate and reduce vehicles and pedestrians density	Visible Light, Scheduling	[179], [205], [206]
Automation	Robot-assisted social distancing	Improve positioning and navigation of robots, especially medical robots inside hospitals	Ultra-wideband, GNSS, Visible Lights, Inertial, Ultrasound,	[149], [177], [178], [209], [210]
	Autonomous delivery systems (e.g., UAVs,$\ldots$)	Reduce the number of people going outside (food, merchandise, etc., delivery)	GNSS, Inertial	[143], [211], [212]
Modeling and Prediction	Infected movement prediction	Predict infected people’s movement	AI	[217]
	Quarantined/at-risk people location prediction	Predict quarantined and at-risk people’s current location to enforce them stay at isolation/protection facility	AI	[218], [219]
	People/traffic density prediction	Predict people density and traffic density	Cellular, AI	[86]–[90], [220]
	Sickness trend prediction	Predict sickness trends in specific areas	AI	[222]

**FIGURE 2.**
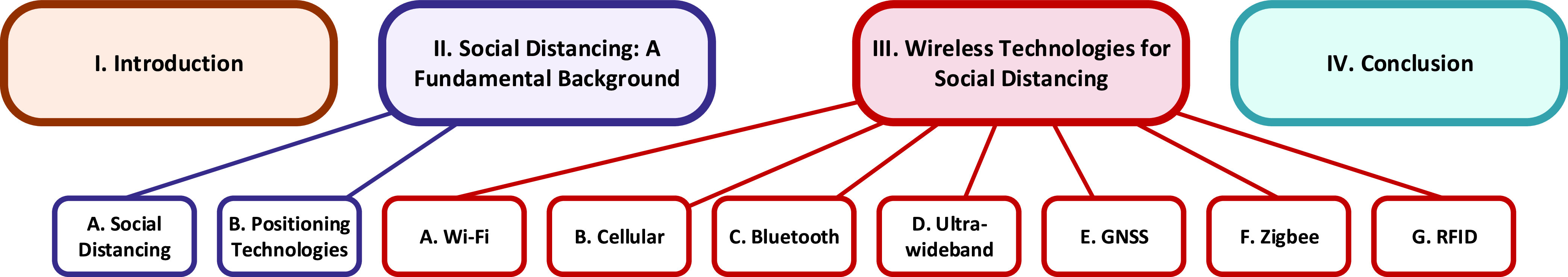
The organization of this (Part I) paper.

As illustrated in Fig. 2, the rest of this paper is organized as follows. We first provide a brief overview of social distancing and distance measurement methods in Section II. Then, Section III discusses enabling wireless technologies for social distancing, and conclusions are given in Section IV.

## II. Social Distancing: A Fundamental Background

### A. Social Distancing

#### 1) Definition and Classifications

Social distancing refers to the non-pharmaceutical measures to reduce the frequency of physical contacts and the contact distances between people during an infectious disease outbreak [11]. Social distancing methods can be classified into public and individual measures. Public measures include closing or reducing access to educational institutions and workplaces, canceling mass gatherings, travel restrictions, border control, and quarantining buildings. Individual measures consist of isolation, quarantine, and encouragement to keep physical distances between people [16]. Although these measures can cause some negative impacts on the economy and individual freedom, they play a crucial role in reducing the severity of a pandemic [11].

#### 2) Measurements and Models

The evaluation of social distancing measures is often based on several standardized approaches. One of the main criteria for social distancing measures selection is the basic reproduction number $R_{o}$ which represents on average how many people a case (i.e., an infectious person) will infect during its entire infectious period [24]. For example, $R_{o}< 1$ indicates that every case will infect fewer than 1 person, and thus the disease is declining in the considered population. Since the value of $R_{o}$ represents how quickly the disease is spreading, $R_{o}$ has been one of the most important indicators for social distancing measures selection [25], [32]. Mathematically, $R_{o}$ can be determined by

\begin{equation*} R_{o}=\int _{0}^{\infty }b(a)F(a)da,\tag{1}\end{equation*}

where $b(a)$ is the average number of new cases an infectious person will infect per unit of time during the infectious period $a$, and $F(a)$ is the probability that the individual will remain infectious during the period $a$ [24].

Besides showing the transmissibility of a disease, $R_{o}$ also gives some intuitive ideas on how to limit the disease spread. As observed from (1), $R_{o}$ can be reduced in different ways, i.e., to decrease $b(a)$ or $F(a)$. To reduce $b(a)$, there are several approaches such as to lower the number of contacts the infected individuals make per unit of time (e.g., avoid mass gatherings and public places closures) or to reduce the probability that a contact will infect a new person (e.g., by wearing masks). To reduce $F(a)$, the infected person needs to be cured or completely avoid contacts with the non-infected (e.g., isolation and quarantine).

For an infectious disease, the most common type of models is the compartmental mathematical model MSEIR [26]. This type of model consists of five main classes M, S, E, I, R which represent different types of individuals in a community as follows:

• M: This class includes the infants with passive immunity passed down from their mothers.
• S: This class represents the susceptible individuals, i.e., people who can become infected.
• E: When an individual of class S is infected, that individual enters a latent period during which it is infected but not yet infectious, i.e., cannot transmit the disease to another individual. The individuals who are in the latent period constitute class E.
• I: After the latent period is over, an individual can transmit the disease to others and is classified by class I.
• R: This class represents the individuals that have recovered from the disease and have infection-acquired immunity.

Different combinations of these classes result in different types of models, such as MSEIR, MSEIRS, SEIR, SEIRS, SIR, and SIRS. The acronyms of these models represent the classes that they take into account and the transition of individuals between these classes. The reason for these variations is that some classes are not needed in certain cases, e.g., birth immunity might not exist for a novel strain of virus. For example, the MSEIRS model is similar to the MSEIR model, except that the recovered individuals can be infected again, and thus the MSEIRS is only used for the cases where immunity is not permanent. Among these models, the classic SIR model is the most common. Let $S(t)$ be the number of susceptible, $I(t)$ be the number of infected, and $R(t)$ be the number of recovered individuals in a population at time $t$. Moreover, let $\beta $ be the average number of adequate contacts (i.e., contacts that infect a new case) and $\frac {1}{\gamma }$ be the average infectious period. Then, the SIR model is defined by

\begin{align*} \dfrac {dS}{dt}=&\dfrac {-\beta I S}{N}, \\ \dfrac {dI}{dt}=&\dfrac {\beta I S}{N}-\dfrac {\gamma I}{N}, \\ \dfrac {dR}{dt}=&\dfrac {\gamma I}{N},\tag{2}\end{align*}

where $N$ is the total population. In (2), since $\beta $ is the number of adequate contacts an infected case made and $S$ is the number of infected cases, $\frac {\beta I S}{N}$ is the rate at which new susceptible individuals are infected. Moreover, the recovery rate is inversely proportional to the infectious period $\frac {1}{\gamma }$, and the total infection rate is the infectious rate minus the recovery rate. It is also worth noting that since $\beta $ is the number of adequate contacts per unit time and $\frac {1}{\gamma }$ is the infection time, $\frac {\beta }{\gamma }$ is the total number of newly infected cases caused by a typical infected individual. This is what $R_{o}$ represents, and thus $\frac {\beta }{\gamma }=R_{o}$.

The abovementioned SIR models neglect several important aspects of a disease such as people with passive immunity and vital dynamics (birth and deaths). Consequently, the model is only effective for modeling a novel strain of an infectious disease (so there is no passive immunity) over a short period of time (birth and death can be neglected). On the other hand, the simplicity of the model ensures that it is well-posed. As a result, the classic SIR model is used in many simulations to predict the infection rate of many infectious diseases.

#### 3) Effectiveness

To evaluate the effectiveness of social distancing, a common approach is to measure the attack rate which is the percentage of infected people in a susceptible population (where no one is immune at the beginning of the disease) at the time of measurement [27]. The attack rate reflects the severity of a disease at a given time, and thus it has different values during the disease outbreak. Among these values, the peak attack rate is often considered and compared to the current healthcare capacity (e.g., intensive care unit capacity) to see the current system’s ability to handle the peak number of patients. After the outbreak is over, data is often collected to determine the final attack rate which is the total number of infected cases over the entire course of the outbreak divided by the total population.

Social distancing measures are proven to be effective when implemented properly [27]–[33]. Different types of social distancing measures may have diverse levels of effectiveness on the disease spread. In [27], the effect of social distancing measures at workplaces is evaluated by an agent-based simulation approach. In particular, six different workplace strategies that reduce the number of workdays are simulated. The results show that, for seasonal influenza ($R_{o}=1.4$), reducing the number of workdays can effectively reduce the final attack rate (e.g., up to 82% if three consecutive workdays are reduced). Nevertheless, in pandemic-level influenza ($R_{o}=2.0$), reducing the number of workdays has a significantly weaker impact, i.e., 3% (one extra day off) to 21% decrease (three extra consecutive days off). Several other studies present similar results. In [28], it is shown that workplace social distancing can reduce the final attack rate by up to 39% in a $R_{o}=1.4$ setting. Similarly, [29] shows that different types of measures can reduce the attack rate from 11% to 20% depending on the frequency of contacts among the employees.

For school closure measures, studies also show positive effects. In [30], a modeling technique is employed to examine the effects of four different social distancing measures under three varying $R_{o}$ settings. Among different types of measures, the school closure measure is shown to be able to reduce the final attack rate by 20%, 10%, and 5%, and the peak attack rate by 77%, 47%, and 32% in the cases where $R_{o}< 1.9$, $2.0 \leq R_{0} \leq 2.4$, and $R_{o}>2.5$, respectively. Similarly, it is shown in [31] that prolonged school closure in a pandemic context can reduce the final attack rate by up to 17% and the peak attack rate by up to 45%.

Another common social distancing measure is the isolation of confirmed cases and cases with similar symptoms. In [32], large-scale epidemic simulations are performed to evaluate different strategies for influenza pandemic mitigation. Among the simulated strategies, the results show that the proper implementation (such that an isolated individual reduces 90% of its contact rate) of isolation can reduce the final attack rate by 7% in a $R_{o}=2$ setting. Similarly, it is shown in [30] that isolation can reduce the final attack rate by 27%, 7%, and 5%, and the peak attack rate by 89%, 72%, and 53% in the cases where $R_{o}< 1.9$, $2.0 \leq R_{0} \leq 2.4$, and $R_{o}>2.5$, respectively.

For household quarantines, studies have shown that this measure can be effective if the compliance level is sufficient. In [32], the effects of voluntary quarantine of household for a duration of 14 days are examined. Simulations are carried out with the assumption that 50% of households will comply, which leads to a 75% reduction of external contact rates, while the internal contact rate will increase by 100%. The results show that this measure can reduce the final attack rate by up to 6% and the peak attack rate by up to 40%. Similarly, in [33], simulations are performed to examine the impacts of different measures. For household quarantines, the result shows that this measure can reduce the final attack rate by 31% and the peak attack rate by 68% with $R_{o}=1.8$ and a compliance rate of 50%.

Apart from the abovementioned measures, the effectiveness of the other social distancing measures either received limited attention or was often considered in combination with another approach. In [32], the effectiveness of travel restrictions and border control measures are examined. However, the results only show that different levels of travel restrictions (from 90% to 99.9%) can delay the peak attack rate by up to six weeks, while how travel restrictions affect the attack rate is not examined. Another type of measure that does not receive much attention is community contact reduction measures (e.g., avoiding crowds and mass gatherings cancellation). In [30], it is shown that this type of measure can reduce the final attack rate by 17%, 14%, and 10%, and the peak attack rate by 72%, 49%, and 38% in the cases where $R_{o}< 1.9$, $2.0 \leq R_{o} \leq 2.4$, and $R_{o}>2.5$, respectively.

When combined together, social distancing measures are proven to be even more effective [30], [32], [34]. It is shown in [30] that when all four measures, i.e., school closure, isolation, workplace nonattendance, and community contact reduction, are in effect, they can drastically reduce the attack rates in all the considered $R_{o}$ settings. In particular, the final attack rate can decrease from 65% to only 3% and the peak attack rate from 474 cases per 10 thousand to only five cases, in the highest $R_{o}$ setting. Similarly, [32] examines the effects when household quarantines, workplace closures, border control, and travel restrictions are combined. The results show that the final and peak attack rates are three times and six times, respectively, lower than when no policy is implemented. Moreover, the peak attack rate can be delayed by nearly three months in a $R_{o}=1.7$ setting. In [34], it is also shown that when four types of measures (i.e., school closure, household quarantines, workplace nonattendance, and community contact reduction) are in effect, the final attack rate can be reduced 3–4 times depending on $R_{o}$.

There are several studies focusing on the negative impacts of social distancing. In [35], simulations are performed based on a standard SIR model to evaluate the benefit and cost of different social distancing strategies. In this study, simulations are carried out without and with social distancing under different caution levels settings. Simulation results are evaluated based on the benefits of the reduced infection rate and the economic cost of reducing contacts. The main finding of this work is that a favorable result can only be obtained by implementing social distancing measures with a high caution level. Since the economic cost is also considered, it is shown that implementing social distancing with an insufficient caution level gives worse results than that of the case without social distancing. In [36], a game theoretical approach based on the classic SIR model is proposed to evaluate the benefits and costs of social distancing measures. Interestingly, the results show that in the case where $R_{o}< 1$, the equilibrium behaviors include no social distancing measures. Moreover, social distancing measures are shown to achieve the highest economic benefit when $R_{o}\approx 2$.

#### 4) Social Distancing in COVID-19

##### a: Protocols and Guidelines

Organizations such as European Centre for Disease Prevention and Control (ECDC), U. S. Centers for Disease Control and Prevention (CDC), U. S. Food and Drug Administration (FDA), Australian Department of Health, and Public Health England have announced various protocols and guidelines [13]–[17] during the current COVID-19 pandemic. Although they are proposed by different organizations, these guidelines and protocols share the same objective to limit the spread of the virus and many similar methods such as keeping physical distance, avoiding mass gatherings, reducing unnecessary physical contact, practicing good hygiene, etc. Generally, these protocols and guidelines vary with the severity of the pandemic at each particular location, e.g., stricter and more detailed protocols are proposed for the places where the pandemic is more severe.

##### b: Effectiveness

**FIGURE 3.**
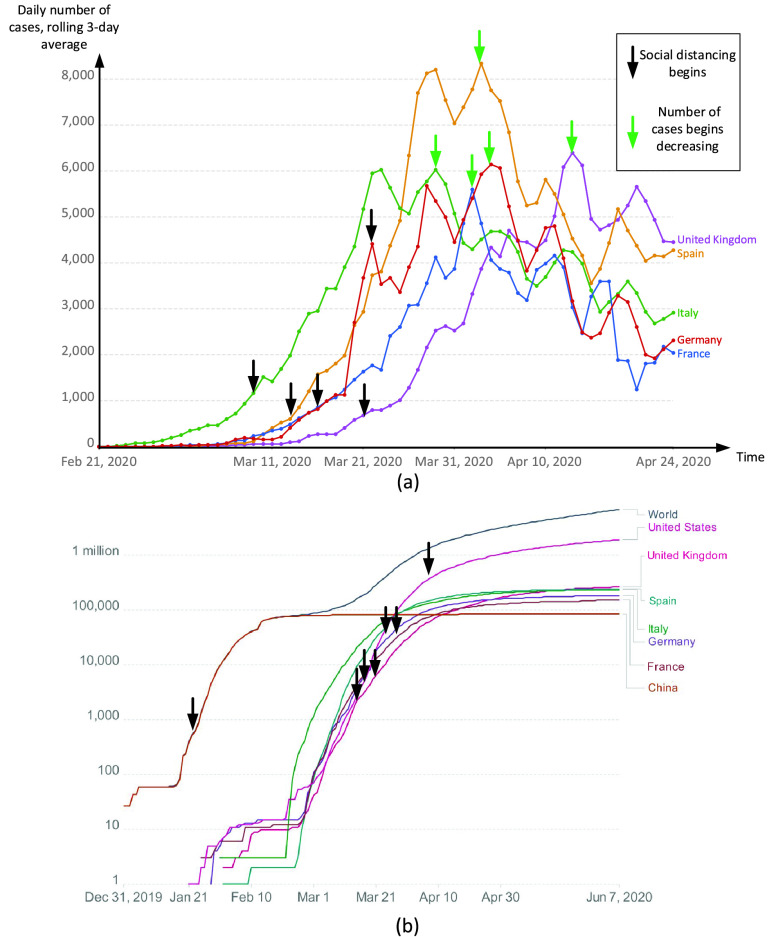
The effects of social distancing in the current COVID-19 pandemic. (a) In several countries, the daily number of cases started to reduce approximately 2–3 weeks after the implementation of social distancing [37]. (b) The impact of social distancing on the total number of cases (flattening the curve) [38].

In the current COVID-19 pandemic, The World Health Organization (WHO) estimates that the value of $R_{o}$ would be in the range of 2–2.5 [46]. As can be seen from the abovementioned studies, social distancing measures can play a vital role in mitigating this pandemic with such $R_{o}$ values. For example, Fig. 3 illustrates the rolling 3-day average of daily new confirmed COVID-19 cases in several countries [37]. Generally, after a country began implementing social distancing (e.g., lockdown at different levels) for 13–23 days, the daily number of new cases begins to drop. As can also be seen from the second graph, the curves representing the total number of cases become less steep after social distancing is implemented (i.e., flattening the curve).

Modeling and simulation approaches also predict positive effects of social distancing on the current pandemic. It is shown in [40] that different combinations of several social distancing measures, including public place closure, self-isolation, household quarantine, and isolation of elderly people, have different effects on the number of cases in ICU. Among them, the combination of all four measures achieves the best effects, i.e., the peak number of cases is nearly 4 times lower and the peak is delayed for three months compared to those of the no social distancing scenario. Moreover, the model also predicts that a second wave will occur in the United Kingdom if social distancing measures are lifted.

Similarly, a prediction modeling approach based on the SEIR model is presented in [41], which shows the effects of different social distancing strategies, including no social distancing, intermittent and continuous social distancing implementation in 16 countries. The simulation predicts that continuous implementation of social distancing achieves the lowest mortality rates in all countries, although the authors suggest that such strategies are not sustainable for low-income countries.

In [42], the authors develop a neural network to study and predict the effects of strict social distancing measures (e.g., quarantine) on the pandemic mitigation in four different regions, namely Wuhan, Italy, South Korea, and the United States. Particularly, the proposed neural network is used to determine the parameters of the SIR and SEIR model, such as $\beta $ and $\gamma $ in (2). The results show that a stricter social distancing strategy has a stronger impact on reducing $R_{o}$, thereby reducing the pandemic severity.

##### c: Challenges

Despite its significant potential, it can be observed that social distancing is very effective only when applied properly. Nevertheless, it is not easy to implement because of many challenges such as:

• *Negative economic impacts*: Many social distancing measures, especially travel restriction, border control, and public places closure have negative impacts on the economy. This may lead to premature lifting of restrictions by the authorities, e.g., Iran, South Korea, China, Germany lifted restrictions too early and had to reimpose restrictions [39], [71].
• *Personal rights violation*: Restriction measures such as quarantines, canceling mass gatherings, and isolation may conflict with ethical and religious principles, e.g., Iran closed religious facilities during lockdown [45]. Moreover, contact tracing and tracking the movement of infected people, e.g., contact tracing in Singapore [105], also violate people’s privacy. Consequently, people might not comply with these measures.
• *Difficulties in changing people’s behavior*: Even when a person wants to comply with social distancing, it is not always easy to do so. For example, it is hard to always keep a safe distance between people (estimating and maintaining a 1.5–2 m distance all the time is not easy), people still have to go outside for healthcare or food, and it is not always possible to work from home (essential workers).
• *Difficulties when many people stay at home*: With the closure of schools and workplaces, many people will have to work or study remotely, which leads to an overwhelming increase in Internet traffic and online service demands, e.g., newly registered users of Zoom [91] and Microsoft Teams [92] have increased 1270% and 775%, respectively, since the lockdown begins.

##### d: Second Waves

These challenges often lead to the premature termination of social distancing measures by the authorities (e.g., lifting restriction) or people (e.g., do not comply with social distancing or resuming normal behaviors too soon) [11]. However, such improper implementation of social distancing may lead to dire consequences such as a second wave of the pandemic (i.e., the attack rate rises sharply again).

As an example, in the previous 1918 influenza pandemic (the Spanish flu), social distancing measures, after their initial success in the first wave, were reduced or ended prematurely by the authorities. Moreover, once the first wave was over, the perceived risk reduced, and people resumed normal behaviors although there had not been an effective pharmaceutical solution yet. These are the main reasons that lead to the second wave [43], [44]. This second wave can be even worse than the first wave, as evidenced by the 1918 pandemic data collected at various geographical locations. For example, in Sydney, the second wave of the Spanish flu pandemic had a slightly higher attack rate but nearly double the mortality rate compared to those of the first wave [43]. Another example is presented in [44], where the mortality data of 16 United States cities were collected during the 1918 influenza pandemic. Among the cities, second waves occurred in 8 cities. Compared to the first waves, the second waves in these cities caused higher death rates in 4 cities and lower death rates in the others.

**FIGURE 4.**
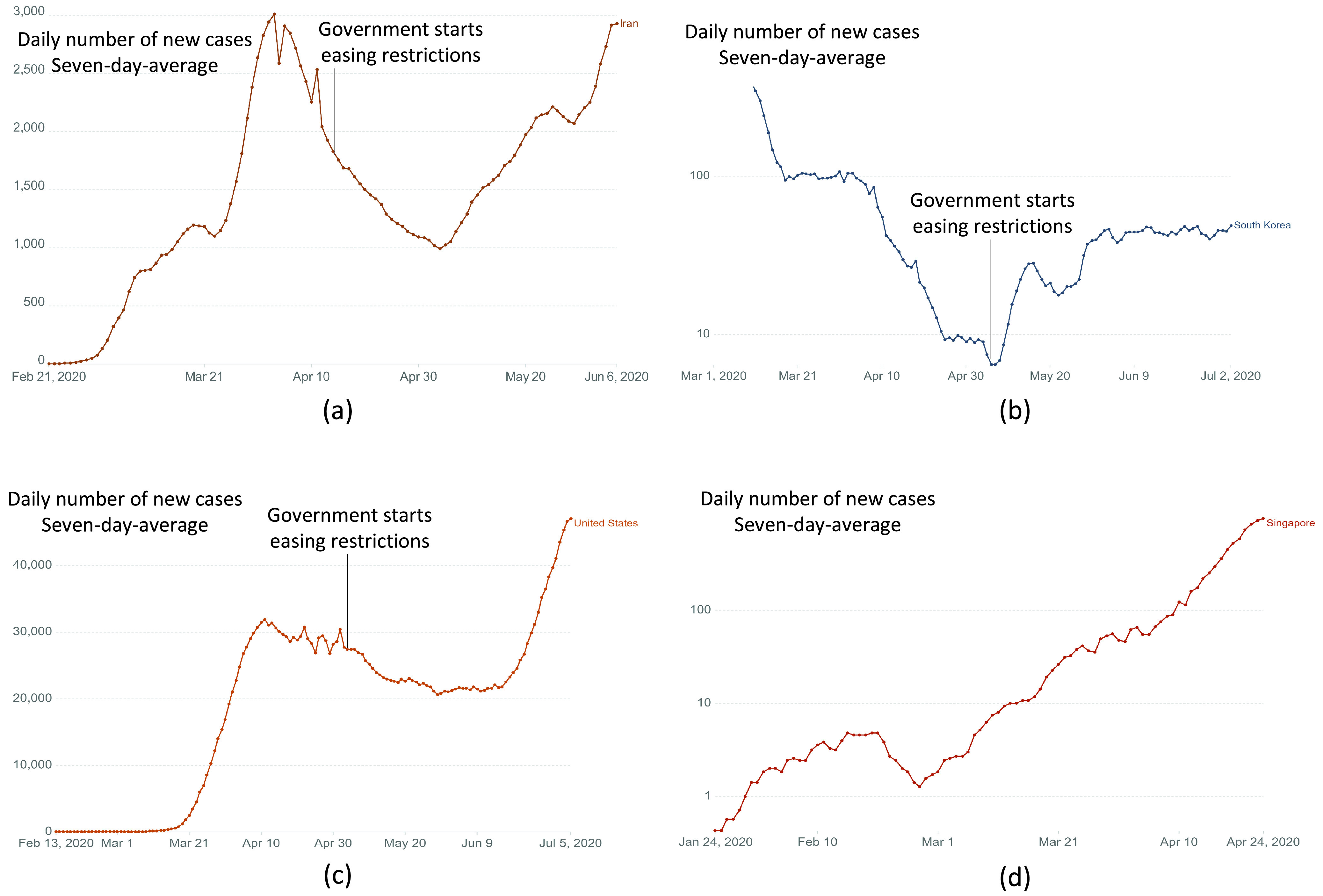
Second waves of COVID-19 in (a) Iran [38], (b) South Korea [38], (c) United States [38], and (d) Singapore [38]. In these countries, the government started easing restrictions too soon, which results in a second wave.

In the current COVID-19 pandemic, several countries, e.g., Iran, South Korea, the United States, and Singapore, have suffered from a second wave as illustrated in Fig. 4. As observed in Fig. 4(a), when the number of new cases began decreasing, the authorities of Iran started to lift and reduce several social distancing measures (e.g., the gradual reopening of government offices, shopping malls, etc.), which led to the second wave after three weeks. Similarly, South Korea and the United States lifted restrictions in early May (e.g., partially reopen bars, restaurants, schools, etc.), and a second wave occurred in both countries shortly after, as shown in Fig. 4(b) and Fig. 4(c), respectively. In the case of Singapore, the authorities did not lift the restrictions prematurely. However, only a partial lockdown was implemented, e.g., schools and businesses were not closed at the beginning. Since social distancing measures were not strictly enforced, its effectiveness relies on people’s perceived risk which decreased as the number of daily new cases decreased. Consequently, the second wave occurred as shown in Fig. 4(d). We can observe from the figures that the pandemic’s severity in the second wave can be much more devastating than that in the first wave, e.g., in the United States and Singapore. Consequently, the authorities have to impose restrictions again, e.g., Iran, South Korea, China, Germany, etc. [39], [71].

Until effective pharmaceutical solutions (e.g., vaccines) are successfully developed and widely available, social distancing remains the best type of measures available to mitigate the pandemic [11]. Therefore, social distancing still plays a vital role in pandemic mitigation, for both the current COVID-19 and future pandemics. In that context, technologies can be leveraged to reduce social distancing negative effects and ensure social distancing proper implementation. Besides technologies, other methods such as creating physical obstacles between people (e.g., plastic dividers, plastic shields, etc. [227]), markings on pavements and roads [228], social distancing awareness signs [228], and law enforcement involvement [228] have been applied to facilitate social distancing. However, these methods are not always available, limited to certain circumstances, and hard to implement on a large scale. Moreover, technologies can be used in conjunction with these methods, e.g., uses technologies to detect crowds and inform law enforcement. Therefore, technologies can play a vital role in facilitating social distancing.

##### e: Practical Scenarios

**FIGURE 5.**
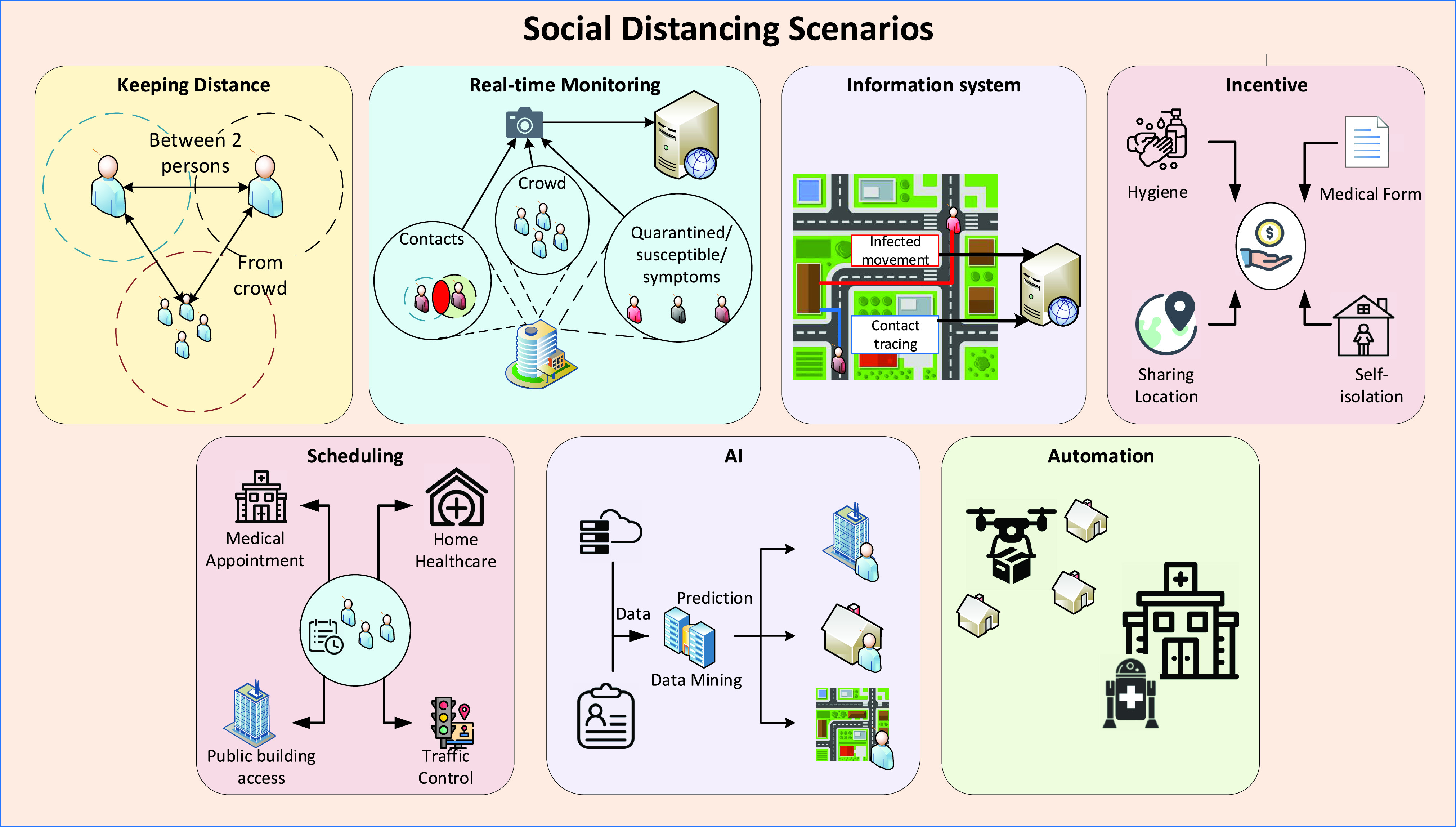
Illustrations of the practical social distancing scenarios identified/proposed in this survey. These scenarios can be categorized into seven main groups: keeping distance, real-time monitoring, information system, incentive, scheduling, AI, and automation.

The practical social distancing scenarios identified/proposed in this survey are categorized and illustrated in Fig. 5. More specific scenarios are summarized in Table 1. The scenarios can be briefly classified as follows:

• *Keeping distance*: In these scenarios, various positioning and AI technologies can assist in keeping sufficient distance (e.g., 1.5m apart) between people. Based on that, when a person gets too close to another or a crowd, the person can be alerted (e.g., by smartphones).
• *Real-time monitoring*: Many wireless and related technologies can be utilized to monitor people and public places in real-time (without compromising citizens’ privacy). The purposes of such monitoring are to gather meaningful data (e.g., numbers of people inside buildings, contacts, symptoms, crowds, and social distancing measures violations) to facilitate social distancing. Based on these data, appropriate measures can be carried out (e.g., limit access to buildings when there are too many people inside, avoid crowds, and alert/penalize violations).
• *Information system*: Technologies such as Bluetooth, Ultra-wideband, Global Navigation Satellite Systems (GNSS), and thermal sensors can be employed to collect the trajectory data of the infected individuals and the contacts that these individuals made. Based on this information, susceptible people who were at the same place or had contacts with the infected ones can take cautious actions (e.g., self-isolation, and test for the disease).
• *Incentive*: Social distancing has negative impacts on personal freedom and the economy. Therefore, incentive mechanisms are needed to encourage people to comply with social distancing measures (e.g., incentivize people to share their movement data and self-isolate). Optimization techniques and technologies such as Bluetooth, Wi-Fi, and cellular together with economics tools like game theory, auctioning, and contract theory can facilitate those incentive mechanisms.
• *Scheduling*: Various scheduling techniques can be employed to increase the efficiency of workforce and home healthcare service scheduling, thereby decreasing the number of employees at workplaces and patients at hospitals. Moreover, scheduling techniques can also be applied for traffic control to reduce the number of vehicles and pedestrians on the street. Furthermore, technologies such as Wi-Fi, Radio frequency identification (RFID), and Zigbee can be applied for building access scheduling.
• *Automation*: In the social distancing context, autonomous vehicles such as medical robots and unmanned aerial vehicle (UAV) can be utilized to reduce the need for human presence in essential tasks, e.g., medical procedures and delivery services. Technologies such as ultra-wideband, GPS, ultrasound, and inertial sensors can be leveraged for the positioning and navigation of these autonomous vehicles.
• *Modeling and Prediction*: AI technologies can be employed for pandemic data mining. The results can help to predict the future trends and movement of the infected and susceptible individuals. Moreover, AI-based classification algorithms can be leveraged to detect disease symptoms in public places.

**FIGURE 6.**
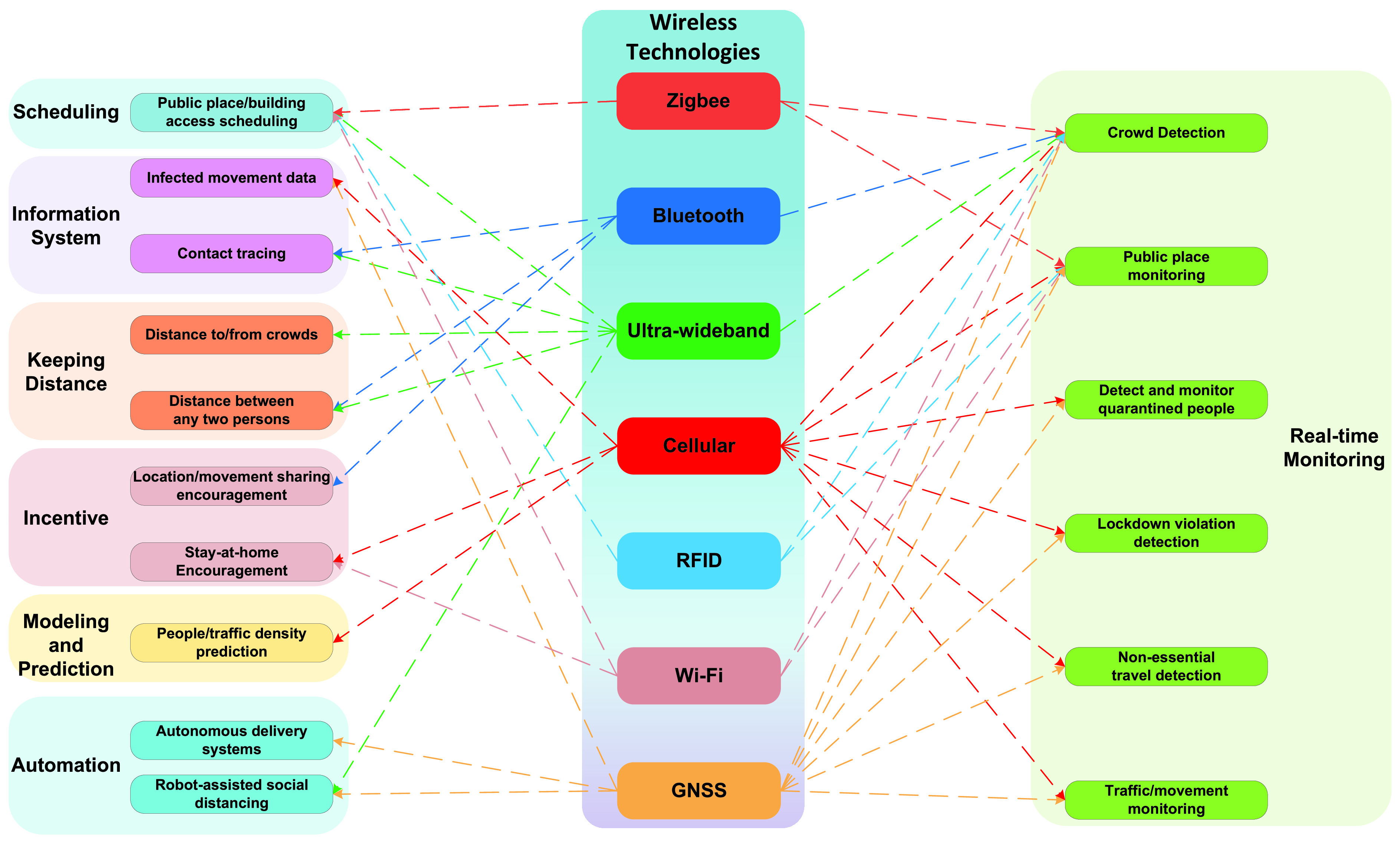
Application of technologies to different social distancing scenarios. Some technologies, e.g., Cellular and GNSS, can be applied to many scenarios, whereas technologies such as Zigbee and RFID are applicable to fewer scenarios due to their limited communication ranges. Scenarios from the same group have the same color. The arrows that show the links from one technology to different scenarios have the same color.

The applications of technologies to specific social distancing scenarios are illustrated in Fig. 6.

### B. Positioning Technologies

Since the main principle of social distancing is to increase the distances of human contacts, approaches to determine the positions and measure the distance between people can play a vital role in facilitating social distancing measures. Using ubiquitous technologies, such as Wi-Fi, cellular, and GNSS, positioning (localization) systems are crucial to many practical social distancing scenarios such as distance keeping, public places monitoring, contact tracing, and automation.

#### 1) Overview of Positioning Systems

**FIGURE 7.**
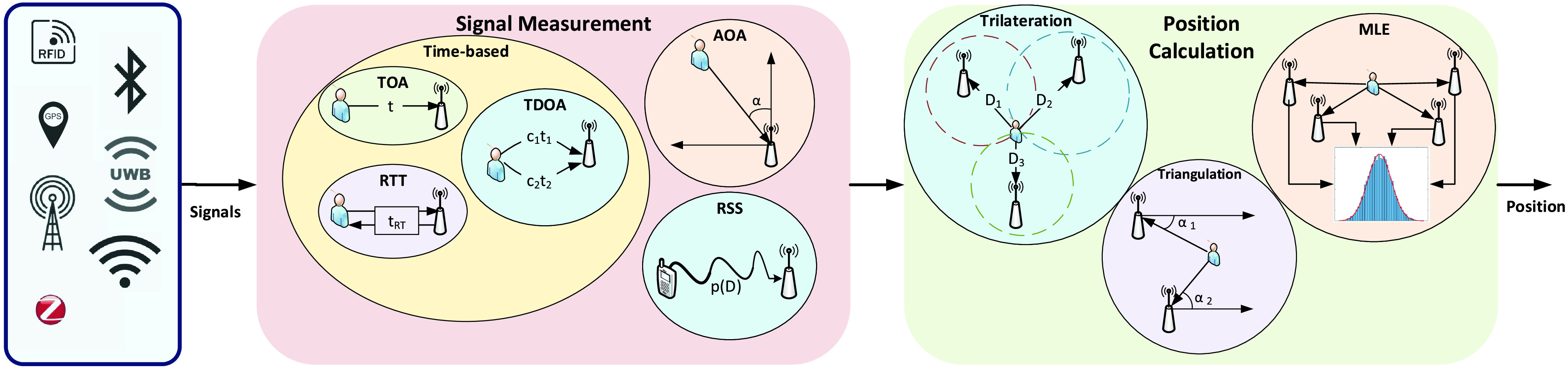
General principle of positioning systems. Signals from sensors are measured using different methods, e.g., time-based, AOA, and RSS, to derive the corresponding signal properties such as traveling times and angles. Based on these measurements, the position of the object can be determined by position calculation techniques such as *trilateration*, *triangulation*, and MLE.

Fig. 7 illustrates the general process and several popular methods of a positioning system [47]. Generally, a positioning system aims to continuously track the position of an object in real-time [23]. To achieve this goal, firstly, signals are transmitted from the target to the receiving nodes (e.g., sensors). From the received signals, useful properties such as arrival time, signal direction, and signal strength (depending on the measurement methods) are extracted in the signal measurement phase. Based on these features, the position of the target can be calculated using various methods in the position calculation phase [47]. Several effective signal measurements and position calculation methods are presented in the rest of this section.

#### 2) Signal Measurements

Typical signal measurement methods can be classified based on the extracted property of the received signal. Among them, time-based methods use the arrival time of the signal to determine the distance between the receiving nodes and the target [47]. Time-based methods can be further classified as follows:

• *Time-of-Arrival (TOA)* [50]: This method determines the distance $D$ between the receiving node and the target based on the time it takes for the signal to travel from the target to the node, i.e.,
  \begin{equation*} D=ct,\tag{3}\end{equation*}
  where $c$ is the speed of the signal transmission and $t$ is the time for the signal to reach the receiving node.
• *Time Difference-of-Arrival (TDOA)* [50]: This method uses two kinds of signal with different speeds and calculates $D$ based on the difference between them, i.e.,
  \begin{equation*} \dfrac {D}{c_{1}}-\dfrac {D}{c_{2}}=t_{1}-t_{2},\tag{4}\end{equation*}
  where $c_{1}$, $c_{2}$, $t_{1}$, and $t_{2}$ are the speeds and arrival time of the two signals, respectively.
• *Round Trip Time (RTT)* [47]: The RTT method measures the duration in which the signal travels to the targets and comes back, i.e.,
  \begin{equation*} D=\dfrac {t_{RT}-\Delta t}{2},\tag{5}\end{equation*}
  where $t_{RT}$ is the time of the whole round trip, and $\Delta t$ is the predetermined delay between when the target receives the signal and when the target starts sending back.

A common disadvantage of the TOA and TDOA methods is that they require synchronized clocks at the node and the target to determine $t, t_{1}$ and $t_{2}$. That may be costly to be implemented as it requires frequent calibrations to maintain accuracy. Although the RTT method does not require clock synchronization, it needs to acquire the delay $\Delta t$ which cannot be predicted in many circumstances [48]. Consequently, extra efforts are needed to determine $\Delta t$.

Unlike the time-based methods, the *Angle-of-Arrival* (AOA) method determines $D$ by measuring the angle of the incoming signals by using directional antennas or array of antennas. The measured angles can then be used in the *triangulation* method to geometrically determine the target position. However, the main disadvantage of this method is that it requires extra directional antennas which are costly to implement [47].

The *Received Signal Strength Indication* (RSSI) method measures the attenuation of the signals to determine the distance. Typically, the relationship between the RSSI and distance can be formulated as follows [53]:

\begin{equation*} P_{R} = \alpha - 10 n \log _{10}(d) + X,\tag{6}\end{equation*}

where $P_{R}$ is the RSSI value at the receiver (e.g., access point), $d$ represents the distance from the user device to the access point, $X$ is a random variable (caused by the shadowing effect) which follows the Gaussian distribution with zero mean. $\alpha $ is a constant value which can be known in advance and depends on fading, antennas gain, and emitted power of the user device. $n$ is the path loss exponent which depends on the channel environment between each user device and the access point. Thus, based on the RSSI level of the received signals, the access point can estimate the position of the user device in indoor environments.

#### 3) Position Calculation

Based on the measured signal properties, different methods are employed to calculate the target’s position. Among them, *Trilateration* is a common method which uses three reference nodes and the distances between them to the target to calculate the position [47], as illustrated in Fig. 7. More specifically, using the coordinates $(x_{1},y_{1}),(x_{2},y_{2}), (x_{3},y_{3})$ of the reference nodes and the corresponding measured distances $D_{1}, D_{2}$, and $D_{3}$, the coordinate $(x,y)$ of the target can be determined by

\begin{align*} \begin{cases} \sqrt {(x_{1}-x)^{2}+(y_{1}-y)^{2}}=D_{1},\\ \sqrt {(x_{2}-x)^{2}+(y_{2}-y)^{2}}=D_{2},\\ \sqrt {(x_{3}-x)^{2}+(y_{3}-y)^{2}}=D_{3}. \end{cases}\tag{7}\end{align*}

Instead of using distances, the *Triangulation* method uses the angles of the signal (from the AOA method) to determine the target’s position. As illustrated in Fig. 7, if the coordinates of two reference nodes and the corresponding measured angles $\alpha _{1}, \alpha _{2}$ are known, the target’s position can be geometrically determined [47].

To address the uncertainty in measurements, the *Maximum Likelihood Estimation* (MLE) method is often employed. This method utilizes the signal measurements from a number of reference nodes (usually three or more) and applies some statistical approaches such as the minimum variance estimation method [49] to calculate the target’s position while minimizing the impact of noises in the environment [47].

## III. Wireless Technologies for Social Distancing

To enable social distancing, many wireless technologies can be adopted such as Wi-Fi, Cellular, Bluetooth, Ultra-wideband, GNSS, Zigbee, and RFID. In this section, we first briefly provide the fundamentals of these technologies and then explain how they can enable, encourage, and enforce people to practice social distancing. After that, we discuss the potential applications, advantages, limitations, and feasibility of these technologies.

### A. Wi-Fi

Due to the fact that Wi-Fi technology is widely deployed in indoor environments, this technology can be considered a promising solution to practice social distancing inside multi-story buildings, airports, alleys, parking garages, and underground locations where GPS and other satellite technologies may not be available or provide low accuracy [20]. In a Wi-Fi system, a wireless transmitter, known as a wireless access point (AP), is required to transmit radio signals to communicate with user devices in its coverage area. Currently, Wi-Fi enabled wireless devices are working according to the IEEE 802.11 standards. Wi-Fi 6 (based on 802.11ax technology) is the latest version of the Wi-Fi standards which provides high-throughput and reliable communications [51]. We discuss a few example scenarios of social distancing that can be enabled by Wi-Fi as follows.

#### 1) Crowd Detection

One potential application of Wi-Fi technology in social distancing is positioning [53], [70]. Based on the location of users, the authorities can detect crowds inside a building and force them to maintain a safe distance. This is an essential factor to practice social distancing during a pandemic outbreak in indoor public places such as train stations and airports. There are two main reasons making Wi-Fi technology possible in social distancing. First, due to the convenience of hardware facilities, we can quickly deploy Wi-Fi systems for user positioning with very low cost and efforts [52]. Second, with recent advances in Wi-Fi-based indoor positioning, Wi-Fi can provide reliable and precise location services to enable social distancing. The most common and easiest way for indoor positioning is to calculate the user’s location based on the RSSI of the received signals from the user device [53], [54]. However, the accuracy of this solution much depends on the propagation model. Thus, in [53], the authors present a new method to dynamically estimate the channel model from the user device to the access point. The key idea of this solution is continuously determining the RSSI values in real-time to obtain the estimated channel model that is close to the real channel model. Once the propagation is estimated, the distance between the access point and the user device can be accurately determined. After that, the user’s location will be derived by using the *trilateration* mechanism.

Differently, the authors in [54] propose to adopt the inertial navigation system (INS) to significantly increase the accuracy of conventional RSSI-based methods. The key idea of this solution is using a Kalman filter to combine and fill the signal database with the INS data. As such, the authors can obtain the average distance error as small as 0.6 m. The above RSSI-based solutions can be easily adopted to detect crowds in indoor environments. Then, the local authorities can take appropriate actions to disperse the crowds or suggest other people not to go to the place. For example, if there are too many people in a supermarket, the authorities can notify and recommend new coming customers to go to other supermarkets or come in another time so that they can avoid crowds.

#### 2) Crowd Detection in Dynamic Environments

Although the RSSI-based solution can detect the user’s location with sufficient accuracy, it may not be effective in dynamic and complicated indoor environments such as airports or train stations [55]–[57]. This is due to the effects of non-line of sight (NLOS) propagation on the wireless signals between the user’s device and the access point, especially in dynamic and complicated environments in which the wireless signals are greatly scattered by obstacle shadows or people (e.g., running and walking) [55]. Another RSSI-based indoor localization technique is the fingerprinting approach (or radio map) that locates devices based on a previously built database. In particular, this database contains the signal fingerprints corresponding to several access points in a specific area. Nevertheless, collecting fingerprint data is time-consuming and laborious [58], especially in large areas such as airports or train stations. In addition, it is infeasible to directly apply the pre-obtained fingerprint database to new areas for localization [59]. The main reason is that the adjustment process to apply the fingerprint database of an area to another is time-consuming and usually requires human intervention.

To address these problems, several solutions [55]–[59] are proposed to enable indoor localization in dynamic and complicated areas such as airports and train stations. With these solutions, the authorities can detect crowds and force people to leave to enable social distancing during pandemic outbreaks. Specifically, in [55], the authors show that when the environment changes, e.g., the presence of people in the line of sight between the user device and the access point, the performance of conventional RSSI-based localization techniques is greatly decreased. Thus, the authors propose an adaptive signal model fingerprinting algorithm to adapt to the dynamic of the environment by detecting users’ positions and updating the database simultaneously. In [59], the authors propose a new localization technique to locate multiple users in different areas by performing a fine-grained localization. In addition, the authors introduce a transfer mechanism to adjust the fingerprint database over multiple areas to minimize human intervention.

An interesting design is proposed in [60] to locate and track people by using Wi-Fi technology, namely Wi-Vi (stands for Wi-Fi Vision). This technology allows the authorities to track people in indoor environments and detect potential crowds, so that they can take appropriate actions to enable social distancing, e.g., inform people not to go to potentially crowded places. In particular, Wi-Vi uses a MIMO interference nulling to remove reflections from static objects and only focuses on moving objects, e.g., a user. Moreover, the authors propose to consider the movement of a user as an antenna array and then track the user by observing its RF beams. If there are many people having the same direction, e.g., going to the same place, the authorities can notify them to avoid forming crowds. Thus, Wi-Vi can be considered a promising technology to enable social distancing.

However, to efficiently detect crowds, Wi-Fi-based localization systems may require several transceivers attached to each access point to obtain high accuracy. Another problem is to differentiate between human and machine terminals. To address this problem, fingerprint databases can be used to detect machine terminals which are usually placed at known locations. Nevertheless, this solution may not be feasible if we consider autonomous robots in the environments, and thus can be a potential research direction.

#### 3) Public Place Monitoring and Access Scheduling

Another way to apply Wi-Fi technology in social distancing is by controlling the number of people inside a building, e.g., supermarket, shopping mall, and university. Specifically, with various Wi-Fi access points implemented inside the building, the number of people currently inside the building can be estimated based on the number of connections from user devices to the access points. For example, the authors in [61] propose a low-cost cyber physical social sensing system which tracks the Wi-Fi messages between the devices, e.g. smartphones, and the access points. Based on these messages, meaningful information such as the number of people within the coverage area of the access points can be extracted. Using this information, several actions can be taken, such as forcing people to queue before entering the building to maintain a safe number of people inside the facilities at the same time. Another application is notifying people who want to go to a building. Specifically, based on the number of people inside the building, the authorities can encourage/force them to stay home or come at a different time if the place is too crowded. However, the accuracy of this approach depends on many factors such as the number of smart devices one person possesses, how many devices can be connected to a network simultaneously, and whether the user connects to the access point as many people completely rely on their cellular connections.

#### 4) Stay-At-Home Encouragement

Wi-Fi technology can also be used to encourage people to stay at home by detecting the frequency of moving outside their houses for a particular time, e.g., a day. Specifically, when user devices move far away from the access point inside their houses, the connection between them will be weak or lost. Based on this information, the access points can estimate the frequency of moving out of their house and then notify the users to encourage them to stay at home as much as possible.

*Summary:* Wi-Fi technology is a prominent solution to quickly and effectively enable, encourage, and force people to practice social distancing. With the current advances of Wi-Fi, the accuracy of localization systems can be significantly improved, resulting in effective and precise applications for social distancing. However, Wi-Fi-based technology is mainly used for indoor environments as this technology requires several access points for localization which may not be feasible for outdoor environments. For outdoor environments, other wireless technologies, e.g., Bluetooth, GPS, and cellular technologies, can be considered.

### B. Cellular

**FIGURE 8.**
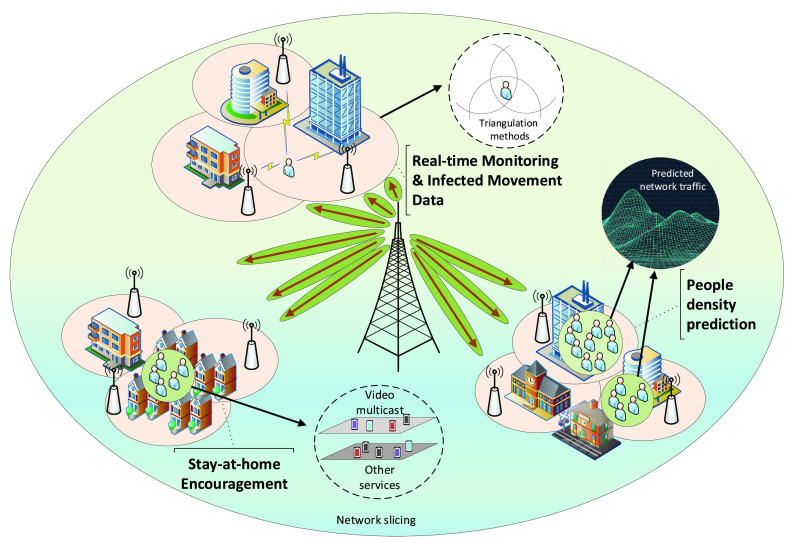
Cellular technology can support different social distancing scenarios. In real-time monitoring and infected movement data scenarios, cellular can help to determine people’s location. Based on these locations, people and traffic density can also be predicted. Cellular can also support Internet-based services, thereby encourage people to stay at home.

Over the past four decades, cellular networks have seen tremendous growth throughout four generations and become the primary way of digital communications. The fifth generation (5G) of cellular networks is coming around 2020 with the first standard. According to the Cisco mobile traffic forecast, there will be more than 13 billion mobile devices connected to the Internet by 2023 [72]. That positions the cellular technology at the center to enable social distancing in many circumstances including real-time monitoring, people density prediction and encouraging stay-at-home by enabling 5G live broadcasting, as illustrated in Fig. 8.

#### 1) Real-Time Monitoring

Individual tracking and mobility pattern monitoring are potential approaches using cellular technology to practice social distancing as shown in Fig. 8(a). According to the 3GPP standard, the current cellular networks, i.e., LTE and LTE-A, are employing various localization methods such as Assisted-GNSS (A-GNSS), Enhanced Cell-ID (E-CID), and Observed TDoA (O-TDoA) as specified in the Release 9; Uplink-TDoA (U-TDoA) included in the Release 11; and with the aids of other technologies like Wi-Fi, Bluetooth, and Terrestrial Beacon System (TBS) as stated in the Release 13 [73], [74]. Cellphone location data collected by the current cellular network is normally used for network operations and managers [74] such as network planning and optimization to enhance the Quality of Service (QoS) rather than user applications due to privacy and network resource concerns. However, in the context of social distancing, user tracking based on data of user movement history can be very effective, e.g., for quarantined people detection, and infected people tracing. The authorities can check whether infected people are violating quarantine requirements or not. In cases they do not follow the requirements, the authorities can send warning messages or even perform some aggressive measures, e.g., fines and arrests, to force them to self-isolate.

Moreover, when a user has been exposed to the virus, the user’s mobility history can be extracted to investigate the spread of the virus. In these cases, the cellular technology can outperform other wireless technologies in term of availability and popularity. For example, localization services relying on wireless technologies such as GPS always need to be run in the foreground application (i.e., the availability), while this service is a part of cellular network operations. In addition, Ultra-wideband and Zigbee technologies require additional hardware [122], [161] (i.e., the popularity). Incoming 5G networks with the presence of key technologies such as mm-Wave communications, D2D communications, and Ultra-dense networks (UDNs) [75] are capable of performing a high precision localization. Two positioning schemes exploiting the mm-Wave communications are proposed in [76] based on the validation of triangulation measurements and angle of differences of arrival (ADoA). The simulation results show that the triangulate-validate and ADoA methods can obtain a sub-meter accuracy level with a probability of 85% and 70%, respectively in a 18 m $\times\,\,16$ m indoor area. The authors in [77] propose a positioning scheme in UDNs using a cascaded Extended Kalman filter (EKF) structure to fuse the DoA and ToA estimations from the reference nodes. The proposed scheme can localize a moving target at speed 50 km/h with a sub-meter level accuracy in an outdoor environment. It can be used for tracking vehicles and monitoring the traffic density.

Recently, some governments have required telecom companies to share cellphone location data to implement social distancing to deal with COVID-19. For instance, Taiwan deployed an “electronic fence” exploiting the cellular-based triangulation methods to ensure that the quarantined cases stay in their homes [78]. The local officials call them twice a day to ensure they do not leave their phones at home and visit them within 15 minutes after their phones are turned off or if they move away from their homes. The Moscow government is also said to be planning to use SIM card data for tracking foreigners and residents who have close contacts with foreigners when the border closure order is lifted [79]. However, individual tracking using cellular technology has raised concerns about privacy [80], [81]. Instead, group/crowd detecting and monitoring based on shared location data which is anonymous and aggregated from carriers become the key approach utilized by several governments such as Italy, Germany, Austria, the UK, Korea, and Australia [82]–[85]. This approach is intended to alleviate privacy concerns compared with individual-level tracking (i.e., it satisfies the EU privacy rules [81]). The metadata can be used to obtain the mobility patterns, and thus the governments can monitor whether people are complying with the lock-down rules or not. It can also be employed to model the spread of the virus to aid the governments in analyzing and evaluating the effectiveness of ongoing quarantine measures during the outbreak.

#### 2) People Density Prediction

In addition to the real-time crowd monitoring and modeling the spread of the virus, the movement history data can be utilized to predict the network traffic due to the large-scale location data provided by carriers and the recent advances of machine learning. There are various works on network traffic prediction proposed in [86]–[90] using the history of users’ movements. Furthermore, the number of users in a specific area can also be estimated from the network traffic of that area as illustrated in Fig. 8(b). Thus, the authorities can predict the crowd gathering in public places (e.g., shopping malls, airports, and train stations) relying on the corresponding forecasted network traffic. Then, appropriate actions can be performed by the authorities to prevent crowd gathering in these places. For example, if the predicted number of people entering a shopping mall exceeds a threshold, the authorities can notify customers to avoid coming to this place at this time or recommend them to go to other shopping malls having lower densities. In addition, this method can also be applied in residential areas to study how often people stay home as well as predict when they go out or the places they come to. This can provide significant data input for network traffic forecasts in public places. In addition, if they regularly go to non-essential places, the authorities can warn or force them to stay at home as much as possible.

#### 3) Stay-At-Home Encouragement

To implement social distancing, many people must do their daily activities remotely from their home such as working, studying, and entertainment. Therefore, some video conference applications used to work from home or study online have witnessed an explosion of downloads. For example, the Zoom application has achieved an increase by 1,270% from 22 Feb to 22 Mar in 2020 [91] and the number of newly registered users of Microsoft Teams has also risen 775% monthly in Italy after the full lock-down was started [92]. As a result, 5G live broadcasting technology can be used to encourage people to stay at home while minimizing the impact on their work, or study (Fig. 8(c)). Especially, this is probably applicable to cases where landline Internet is not available. There are many works to enhance the quality of video multicast/broadcast applications by utilizing the advances of 5G networks [93]–[97]. Video multicast/broadcast services are defined as an ultra-high definition slice in a MIMO system [93]. To improve the spectral efficiency for video multicast/broadcast in the proposed system, the authors introduce a hybrid digital-analog scheme to tackle channel condition and antenna heterogeneity. Another possible solution that can significantly improve qualities for video multicasting/broadcasting is data caching. A novel caching paradigm proposed in [94] is applied for multicast services in heterogeneous networks. With the awareness of multicast files, the proposed caching policy can select files efficiently for the caches. Studies in [95], [96] propose using NOMA techniques to support multicast/broadcast by increasing the spectrum efficiency in multi-user environments. Finally, the authors in [97] propose a video multicast orchestration scheme for 5G UDNs which can help to improve the spectrum efficiency.

#### 4) Infected Movement Data

Due to the omnipresence of mobile phones and the near world-wide coverage of cellular signals, cellular technology can be an effective tool to track the movement of people. Unlike in the quarantined people detection scenarios where these people may deliberately leave their phones at home, people do not have any reason to do so in the infected movement data scenario. Therefore, cellular can be an effective technology in this scenario. The authors of [190] summarize the methods to trace human position in outdoor environments using base stations and indoor environments using access points. However, the positioning accuracy for outdoor environments still needs to be improved because a small error by using the cellular network technology can cause a big error in the distance measurement.

*Summary:* Cellular technology can be considered one of the most important approaches to assist social distancing. It can be deployed on a large scale due to its convenience and omnipresence compared to other wireless technologies. It can be used to track quarantined or infected individuals. Furthermore, it can provide a unique solution to not only monitor crowds in real-time, but also allow the local authorities to predict the forming of crowds in public areas (e.g., airports, train stations, and shopping malls) based on the forecasted network traffic. The low latency feature of 5G networks in data processing using edge/fog computing enables quick responses of the authorities (e.g., send notifications instantly), for example, to prevent close contact. However, the use of subscriber’s location data for social distancing measures is subject to great privacy concerns from citizens.

### C. Bluetooth

With the explosive growth of Bluetooth-enabled devices, Bluetooth technology is another solution for social distancing in both indoor and outdoor environments. In particular, Bluetooth is a wireless technology used for short-range wireless communications in the range from 2.4 to 2.485 GHz [98], [99]. Bluetooth devices can automatically detect and connect to other devices nearby, forming a kind of ad-hoc called piconet [99]. Recently, Bluetooth Low Energy (BLE) has been introduced as an extended version of the classic Bluetooth to reduce the energy usage of devices and improve the communication performance [99]. Given the above, the BLE localization technology possesses several advantages compared with those of the Wi-Fi localization. First, the BLE signals have a higher sample rate than that of the Wi-Fi signals (i.e., 0.25 Hz ~ 2 Hz) [100]. Second, the BLE technology consumes less power than that of the Wi-Fi technology, and thus it can be implemented widely in handheld devices. Third, the BLE signals can be obtained from most smart devices, while Wi-Fi signals can be obtained from only access points. Finally, BLE beacons are usually powered by battery, and thereby they are more flexible and easier to deploy than Wi-Fi. It is worth noting that Bluetooth is mainly used for infrastructureless adhoc communications in contrast to other technologies.

#### 1) Contact Tracing

**FIGURE 9.**
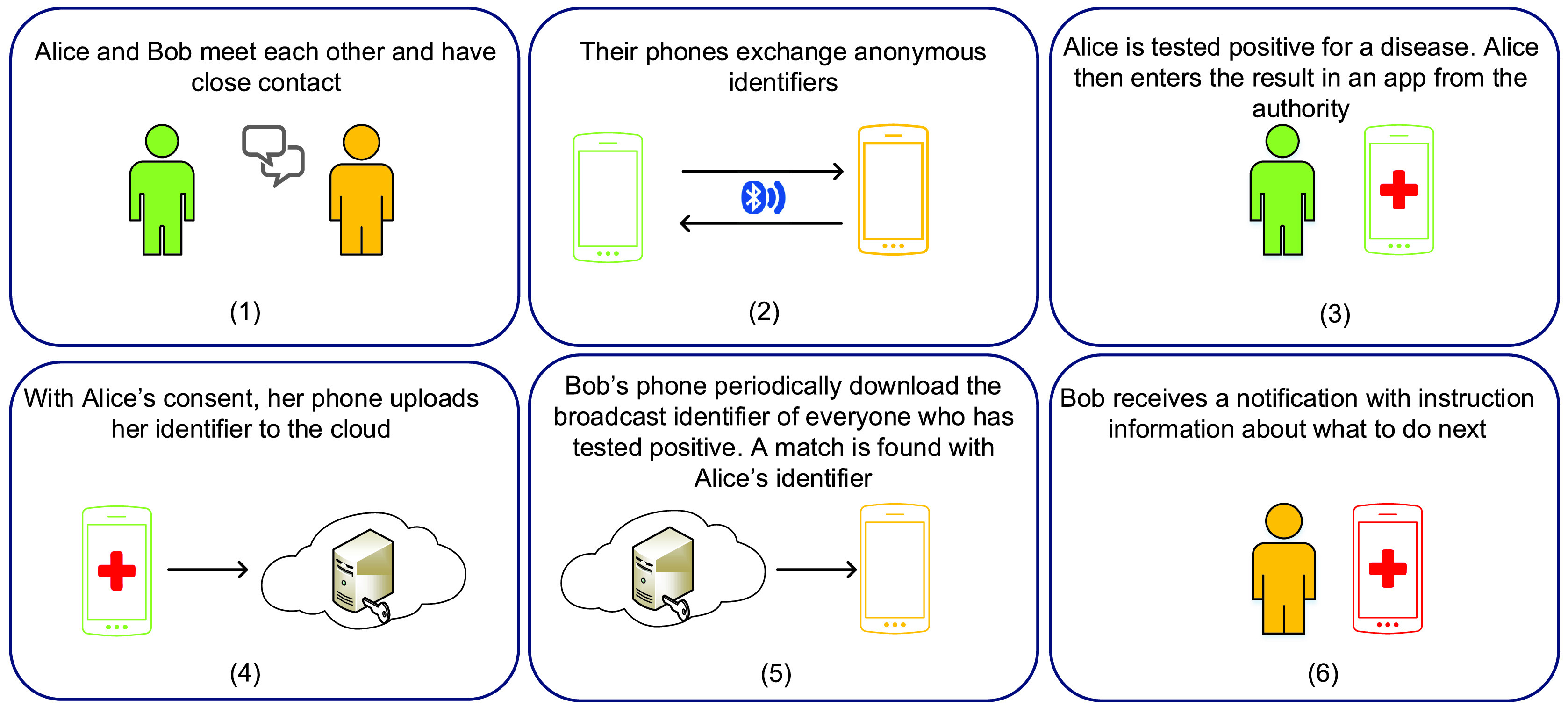
Contact tracing application based on Bluetooth technology [103]. The application will record the event when two people have close contact with each other. Later, when one of them is tested positive for an infectious disease, the application can notify the other person.

One application of Bluetooth in social distancing is contact tracing [101], [102] as illustrated in Fig. 9. The key idea is using Bluetooth to detect other users in close proximity with their information (e.g., identifier) stored in a person’s Bluetooth device, e.g., a mobile phone. When there is an infected case, the authorities can ask people to share these records as a part of a contact tracing investigation. Thereby, the authorities can detect people who may have close contact with the infected one and notify them promptly to prevent the spreading of diseases. Several attempts to use Bluetooth in contact tracing have been reported. Apple and Google have recently introduced a mobile application (running on both iPhone and Android devices) that can detect other smartphones nearby using Bluetooth technology [103]. If a person is tested positive for a disease, he/she will enter the result in the app to inform others about that. Then, people who may have close contact with the positive case will be notified and instructed about what to do next. Note that a Wi-Fi or cellular connection would also be required to enable the app. Similar apps have been recently launched in Singapore [105], Europe [107], and India [108].

#### 2) Crowd Detection

Bluetooth technology can be used to detect crowds in indoor environments to practice social distancing with the latest advances in Bluetooth localization techniques [111], [113]. In particular, based on signals received from users’ Bluetooth devices, a central controller can calculate the positions of users and detect/predict crowds in indoor environments. If a crowd is detected, the local manager can force people to leave to practice social distancing. In addition, they can advise people who want to go to the place to come at a different time if the place is too crowded at the moment. In [111], the authors point out that with the development of BLE, Bluetooth-based indoor localization can be considered a practical method to locate Bluetooth devices in indoor environments due to its low battery cost and high communication performance. The authors then propose indoor localization schemes that collect RSSI measurements to detect the user’s location by using the triangulation mechanism.

In [112], the authors show that the BLE technology is strongly affected by the fast fading and interference, resulting in a low accuracy when detecting the user’s device. To improve the accuracy of the BLE positioning, the authors run several experimental tests to choose the optimal parameters to set up BLE localization systems. The authors demonstrate that the BLE-based indoor localization can achieve a better performance than that of Wi-Fi localization systems. The authors of [114] point out that the accuracy of BLE-based localization is strongly affected by advertising channels, human movements, and human obstacles. To address these problems, they propose a dynamic AI model that can detect human obstacles by using three BLE advertising channels. Then, the RSSI values will be compensated accordingly.

In [115] and [116], the authors show that Wi-Fi-based and Bluetooth based localization systems can be strongly affected by the interference from other wireless devices operating at 2.4 GHz bands. To mitigate the interference, Wi-Fi devices can use 802.11b and 802.11g/n standards which deploy direct-sequence spread spectrum and orthogonal frequency-division multiplexing signaling methods. Similarly, Bluetooth devices can avoid interference from other wireless devices, e.g., Wi-Fi enabled devices, by using the spread-spectrum frequency hopping technique to randomly use one of 79 different frequencies in Bluetooth bands. As such, the interference from other devices is significantly reduced, thereby improving the accuracy of localization systems.

#### 3) Distance Between Two People

**FIGURE 10.**
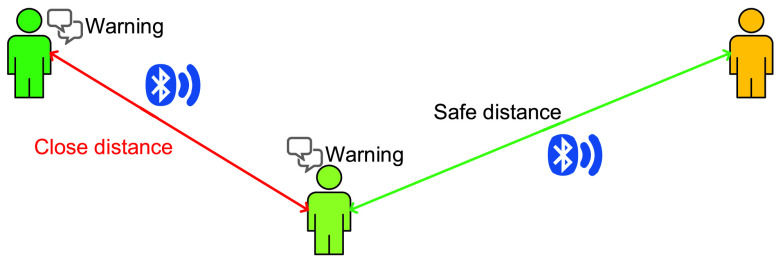
Keeping distance between any two persons using Bluetooth technology. A Bluetooth-enabled device such as a smartphone can calculate the distances between itself and other nearby Bluetooth-enabled devices. When another device comes into close proximity, a warning notification can be sent to the user.

Bluetooth can also be used to determine the distance between two persons by using their Bluetooth-enabled devices, e.g., smartphone or smartwatch, as depicted in Fig. 10. Specifically, similar to the Wi-Fi technology, based on RSSI levels, a device can calculate the distances between itself and other nearby devices [113]. It is worth noting that Bluetooth technology can allow a device to connect to multiple devices at the same time [98]. Thus, the device can simultaneously detect distances to multiple devices in its coverage. If the distance is less than a given threshold, e.g. 1.5 meters [13], the devices can warn and/or encourage users to practice social distancing.

*Summary:* Bluetooth technology is a very promising solution to enable social distancing. However, the privacy of users needs to be taken into account as the applications require users to share information with the authorities and third parties. This can be a research direction to ensure privacy and encourage people to share their information to prevent the spreading of diseases. In addition, there are several drawbacks of Bluetooth technology in social distancing which need to be considered such as the accuracy of localization techniques when the users’ devices are located inside the pockets or bags and their devices always need to turn on the Bluetooth mode. Furthermore, combining Bluetooth and other technologies (e.g., Wi-Fi [117]) to improve the localization accuracy is also an open research direction.

### D. Ultra-Wideband

Ultra-WideBand (UWB) technology has been deemed to be a promising candidate for precise Indoor Positioning Systems (IPSs) that can sustain an accuracy at the centimeter level in the ranges from short to medium. This is due to its unique characteristics (e.g., high time-domain resolution, immunity of multipath, low-cost implementation, low power consumption, and good penetration) [118]. Due to the wide bandwidth nature of UWB signals (at least 500 MHz as specified by FCC [119]), the impulse radio (IR) UWB technology has the capability of generating a series of very short duration Gaussian pulses in time-domain which enables its advantages compared with other RF technology. Pulse position modulation with *time hopping* (TH-PPM) is the most popular modulation scheme exploited in the impulse radio based UWB [120]. This pulse can directly propagate in the radio channel without requiring additional carrier modulation. The baseband-like architecture of the IR-UWB facilitates extremely simple and low-power transmitters. Thus, the advantages of the IR-UWB technology can greatly support social distancing, even better than other wireless technologies (e.g., higher accuracy in indoor positioning applications) or provide exclusive solutions (e.g., device-free tracking/counting) for some scenarios, as discussed below.

#### 1) Real-Time Monitoring

In this section, we review some social distancing scenarios using Ultra-wideband technology for real-time monitoring such as crowd detection (e.g., tracking users’ location), public place monitoring and access scheduling (e.g., counting the number of people in a specific area).

##### a: Crowd Detection

One of the major solutions for crowd detection is tracking locations of people in public areas. There are many commercial products exploiting the IR-UWB technology for real-time localization in both daily life and factories such as DecaWave [121], BeSpoon [122], Zebra [123], Ubisense [124]. DecaWave and BeSpoon claim their products based on ranging measurements can offer an accuracy under 10 cm [121], [122]. Furthermore, Ubisense and Zebra provide industrial products which can obtain a high accuracy even in cluttered, indoor factory environments [123], [124]. All of them support real-time positioning for multiple mobile tags by using the triangulation techniques based on the absolute locations of reference nodes or anchors (e.g., UWB transceivers). Especially, the Dimension4 sensor invented by Ubisense can be integrated with a built-in GPS module for outdoor tracking purposes. Experiments conducted to evaluate holistically the performance of three commercial products (i.e., DecaWave, BeSpoon, and Ubisense) under indoor industrial environment setting (with the presence of NLOS) can be found in [125]. The availability of commercial UWB-based localization systems enables real-time people tracking in public places by localizing their UWB-supported phones, or personal belongings equipped with tags (e.g., keys and shoes). Thus, the authorities can detect the crowd to notify them and other people in the area, disperse the crowd or even predict and prevent the forming of the crowd by using AI/Machine learning algorithms based on the previously collected data.

Recently, device-free localization (or passive positioning) techniques have witnessed significant interest. This is due to the capability to tackle inherent problems of aforementioned communication-based localization approaches: (i) privacy issues (e.g., tracking targets do not need to communicate with an access point/network coordinator, and thus it can protect private information of the target), and (ii) physical obstacles (e.g., LOS communications have significant impact by obstacles) [126]. The high time-domain resolution feature of the IR-UWB technology enables the device-free localization methods relying on the changes of very short pulses properties between two transceivers because of absorption, scattering, diffraction, reflection, and refraction [127], [128]. In particular, the authors in [127] use monostatic radar modules (i.e., P410 platform) equipped with one transmitter and one receiver for multi-target tracking based on Gaussian mixture probability hypothesis density (GM-PHD) filters. Information (including raw signal, bandpass signal, motion filtered signal, and detection list) extracted from the reflected signals is used to estimate the locations of targets with an accuracy at the decimeter level. To improve the accuracy, a multi-static is deployed in [128] to track a person in real-time by determining the difference between the channel impulse response with the presence of a new object and that of the previous one without the object. The location of the object can be found with the mean error of only 3 cm by applying a leading edge detection algorithm on the difference between the two measurements. However, the limitation of this work is that it can track only one target at a time. Motivated by the above works, we can easily deploy device-free localization techniques for crowd detection in public areas without revealing any personal information and hardware requirements on target objects. Thereby, the authorities can locate the exact locations of crowds and have appropriate actions to disperse crowds or force them to practice social distancing.

##### b: Public Place Monitoring and Access Scheduling

**FIGURE 11.**
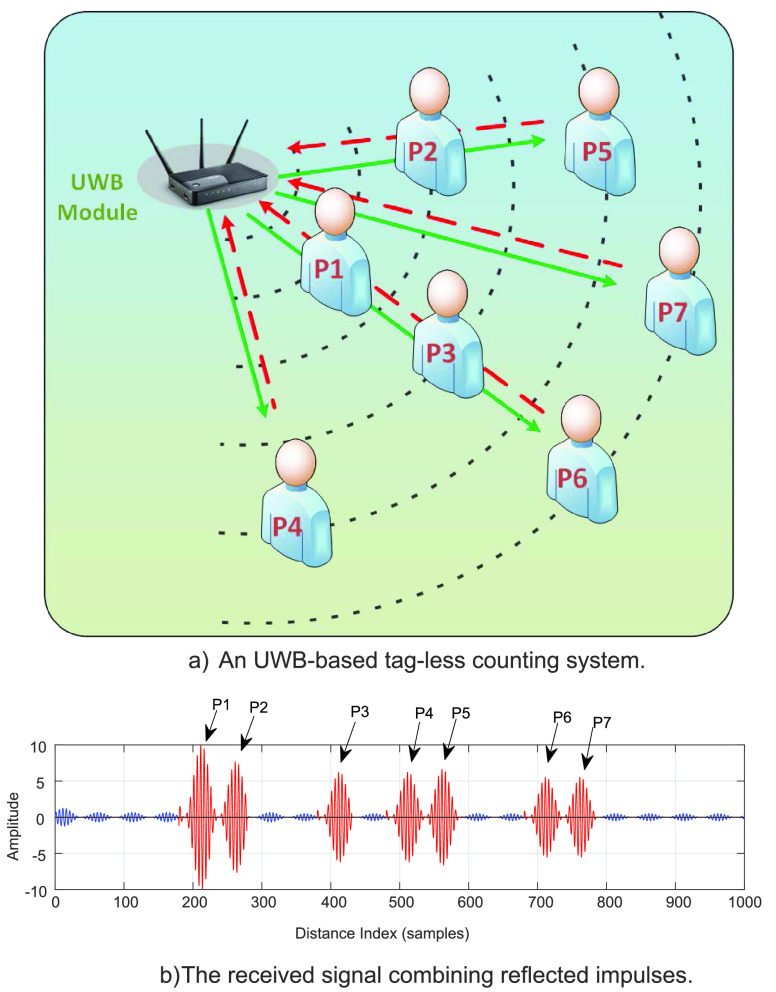
Tag-less counting technique using the UWB technology. (a) The UWB module emits an impulse signal and receives different signals reflected back from people and other objects. (b) Signals reflected from people (red) and objects (blue) have different amplitudes. Using a maximum likelihood approach, the number of people can be determined.

A simple solution for public place monitoring is referred to as device (or tag)-free counting techniques [129], [130]. Specifically, the authors in [129] propose an advanced people counting algorithm using the revelation of the received signal pattern according to the number of people as illustrated in Fig. 11(a). This method enables people counting even with the presence of dense multipath signals in the environment which is not able to be performed by counting techniques based on detecting single signals corresponding to individual persons. For example, other counting approaches using Wi-Fi and Zigbee rely on the number of connections from users to an access point (i.e., Wi-Fi) or a network coordinator (i.e., Zigbee). Major clusters are picked up to find main pulses having maximum amplitudes. A joint probability density function derived from these main pulses is utilized to derive the maximum likelihood (ML) equation. Then, the estimated number of people is determined to be the figure having the maximum likelihood as shown in Fig. 11(b). Similarly, the solution in [130] also provides a counting approach without positioning targets by using the *crowd-centric* method based on energy detection. Without requiring hardware deployment like Wi-Fi and Zigbee, the approaches proposed in [129], [130] can provide a low-cost and high-privacy solution to detect the number of people in public areas. Further actions can be conducted by the local manager to maintain social distancing such as scheduling people to enter the place based on the counting information or giving advice to other people who are planning to go to the crowded place to come at a different time.

#### 2) Keeping Distance and Contact Tracing

Similar to Bluetooth, the IR-UWB technology can also be applied to maintain the distances between people as well as close physical contact tracing by using ranging methods with high precision in both indoor and outdoor environments [121], [131]. While DecaWave provides a ranging measurement using sensors and tags [121], Apple has already brought this feature to their phones (e.g., Iphone 11 series) for their primitive location-based services (e.g., finding objects and improving AirDrop) [131]. These approaches use time-based ranging techniques like ToF, TDoA or combined ToF and AoA to measure the distances to nearby sensors, tags, or phones. However, these products can be employed to detect close proximity between users in public places. Thanks to the IR-UWB technology, they can frequently broadcast pilot messages containing some information (e.g., their specific IDs, timestamp, etc.) to nearby devices for ranging measurements with extremely low energy consumption. Then, surrounding devices can utilize the information of the received messages to estimate the distance from the source device and warn the users if they are too close to each other (e.g., less than 1.5m in a pandemic situation [13]). In addition, these devices can also store other information like who had close contacts with them along with the distances and duration periods. This information is very important because it can be used to trace close contacts in the future (e.g., investigate the spread of the virus in a pandemic) with minimal privacy violation.

In order to help people to avoid crowds, especially vulnerable or at-risk groups, in indoor environments such as shopping malls, hospitals, and office buildings, BeSpoon introduces a commercial product that allows moving targets to self-localize their positions very accurately (i.e., less than 10 cm over 600 m in LOS environments) in a short time by using the IR-UWB technology [122]. This product provides both evaluation kits and an ultra-compact UWB module which can be easily integrated into off-the-shelf products (e.g., shopping trolleys or baskets) for localization and navigation purposes. A SnapLoc platform proposed in [132] allows an unlimited number of tags to self-estimate their locations at position update rates up to 2.3 kHz. It uses the TDoA technique based on all simultaneous responded information from reference nodes integrated into one single channel impulse response. By combining with the positioning service (i.e., to provide locations of other people in a specific area), a navigation application exploiting commercial products like BeSpoon can be developed to assist people (e.g., customers) for self-detecting their current locations as well as crowds’ locations along the way, thereby assisting them to plan their moves and navigate to stay away from crowds.

*Summary:* With the aforementioned potential applications, the IR-UWB systems can be considered an outstanding solution to handle social distancing in both indoor and outdoor environments. The IR-UWB based localization systems discussed in [121]–[124] can be employed for detecting and monitoring crowds in public places with a low-cost deployment. Although this technology can also be used to monitor the positions of self-isolated people to check whether they may violate the quarantine requirements or not, it is less attractive than other RF technologies like Wi-Fi or cellular which do not require to install additional hardware for tracking purposes. In addition, UWB-enabled phones like iPhone 11 series can assist users in practicing social distancing without localization and navigation services. However, this solution only works with a modern iPhone equipped with a UWB chip. Last but not least, the device-free technology presented in [127]–[130] is a great advantage of the IR-UWB technology compared with other wireless technologies for the crowd detecting and monitoring in public places with acceptable accuracy at the decimeter level [127].

### E. Global Navigation Satellite Systems (GNSS)

The GNSS has been being the most widely used for positioning purposes in the outdoor environment nowadays. GNSS satellites orbit the Earth and continuously broadcast navigation messages. When a receiver receives the navigation messages from the satellites, it calculates the distances from its location to the satellites based on the transmitting time in the messages. Basically, to calculate the current location of a user, it requires at least three different navigation messages from three different satellites (based on the *Trilateration* mechanism in Section II). However, in practice, to achieve high accuracy in calculating the location of a user, at least four different messages from four satellites are required (the fourth one is to address the time synchronization problem at the receiver) [133]. Currently, some GNSS systems (e.g., Galileo [134]) can achieve an accuracy of less than 1 m. As a result, GNSS systems can be considered a very promising solution to enable social distancing practice.

#### 1) Real-Time Monitoring

Due to the outstanding features of GNSS technology in locating people, especially in outdoor environments, this technology is very useful for tracking people to practice social distancing. Specifically, most smartphones are currently equipped with GPS devices which can be used to track locations of mobile users when needed. In the context of a pandemic outbreak, e.g., COVID-19, people suspected of being infected, for example, returning from an infected area, will be required to be self-isolated. Thus, to monitor these people, the authorities can ask them to wear GPS-based positioning devices to make sure that they do not leave their residences during the quarantine [146], [147]. The main advantage of using GNSS technology compared to Wi-Fi or Infrared-based solutions for people tracking is that this technology allows to monitor people anywhere and anytime globally, and thus even if the suspects move from one city to another city, the authorities still can track and monitor them. However, one of the major disadvantages of this technology is that it depends on the satellite signals. Thus, in some areas with weak or high interference signals (e.g., inside a building or in crowded areas), the location accuracy is very low [140], [144], [148]. To overcome this limitation, pseudolites have been proposed. Pseudolites are ground-based transceivers that can act as an alternative for satellites to transmit GNSS signals. These pseudolites can be installed in the areas where satellite signals are weak to enhance the positioning accuracy of the GPS. Nevertheless, pseudolites have not been widely deployed because of their high price and strict time synchronization requirement [145].

#### 2) Automation

Another useful application of GNSS to practice social distancing is automation. It comes from the fact that GNSS is especially important for navigation in autonomous systems, such as robots, UAVs, and self-driving cars. Thus, in a pandemic outbreak when people are required to stay at home, GNSS-based autonomous services play a key role to minimize physical contact between people. For example, customers can shop online and receive their items with drone delivery services. Such kind of services has been introduced recently by some large retail corporations such as Amazon and DHL. Similarly, robotaxi services have been introduced recently in some countries to deal with COVID-19 outbreak [143], [149]. It can be clearly seen that these GNSS-based autonomous services can contribute a significant part in implementing social distancing in practice by minimizing the required human presence for delivery and transportation.

#### 3) Keeping Distance and Crowd Detection

In [142], the authors introduce a GNSS service which can be used to determine the locations of users, thereby being able to warn them if they violate the social distancing requirements. In particular, in this service, mobile users are required to install a mobile application which can track the location of the users based on GPS technology. Then, the users’ locations will be updated constantly to the service provider. Thus, based on the users’ locations, the service provider can determine whether the user violates the social distancing requirements or not. For example, if there are more than two users located too close to each other (e.g., less than two meters), the service provider can send warning messages to remind the users. Furthermore, in the cases where a user goes to restricted areas, e.g., isolated areas, they will receive warning messages to be aware of using protection measures.

#### 4) Infected Movement Data

In the infected movement data scenario, GNSS can be a very effective technology because of its world-wide coverage and positioning accuracy is not the main concern. For the outdoor environment, using GNSS alone can be sufficient for tracking the location of infected people. With the omnipresence of smartphones with built-in GPS feature, the movement path of the infected people can be easily determined. However, the main concern in this scenario is that people have to turn on GPS service on their smartphones, which necessitate mechanisms to incentivize people to share their movement information.

*Summary:* Although this GNSS-based service has many advantages in practicing social distancing, e.g., tracking users, keeping distance, and group monitoring, it has some shortcomings which limit its applications in practice. Specifically, this service requires tracking locations of users based on GPS in a real-time manner, which may cause some extra implementation costs and privacy issues for users. Furthermore, in terms of determining the distance between two people, the accuracy of GNSS services is not high in general, especially for distances less than two meters. Thus, some recent advanced GNSS technologies like [136], [138], [139], [145] can be used to improve the accuracy of the GPS. However, these technologies are still quite expensive and have not been widely deployed for public services, and thus more research in this direction should be further explored. Privacy issues will be discussed in Part II [1] with several solutions such as location information protection and personal identity protection.

### F. Zigbee

Zigbee is also a potential technology that can help to enable social distancing. In particular, Zigbee is a standard-based wireless communication technology for low-cost and low-power wireless networks such as wireless sensor networks. A Zigbee system consists of a central hub, e.g., network coordinator, and Zigbee-enabled devices. Zigbee-enabled devices can communicate with each other at the range of up to 65 feet (20 meters) with an unlimited number of hops. Compared with Wi-Fi and Bluetooth technologies, Zigbee is designed to be cheaper and simpler, making it possible for low-cost and low-power communications for smart devices [159], [160]. Moreover, Zigbee can operate at several frequencies, such as 2.4 GHz, 868 MHz, and 915 MHz. Given the above, Zigbee is ideal for constructing mesh networks with long battery life and reliable communications [160]. As a result, Zigbee can be considered a promising candidate in several applications that enable social distancing during a pandemic outbreak.

#### 1) Crowd Detection

One promising application of Zigbee is detecting and tracking users’ location in indoor environments. The key idea is that based on the RSSI level of the received signals from the user’s Zigbee-enabled device, the Zigbee control hub can determine the location of the user. Several research works report that Zigbee localization systems can achieve high accuracy with low-power and low-cost devices [159]. Based on the location of users, the central hub can detect crowds, i.e., many users in the same area, and notify the local manager to ask people to practice social distancing during a pandemic outbreak. With the state-of-the-art mechanisms in the literature, the accuracy of Zigbee localization systems is significantly improved, making it feasible for social distancing. In [161], the authors propose a novel framework to enhance the localization accuracy of Zigbee devices by considering the effect of “drift phenomenon” when users move from one place to another place in indoor environments. The authors then demonstrate that the proposed framework can increase the accuracy by up to 60% compared with conventional solutions.

Differently, in [162], the authors introduce an ensemble mechanism to further improve the localization accuracy. In particular, instead of using the RSSI level, the proposed solution combines the gradient-based search, the linear least square approximation, and multidimensional scaling methods together with spatial dependent weights of the environment to approximate the target’s location. In [163], the authors propose an energy-efficient indoor localization system that can obtain Wi-Fi fingerprints by using ZigBee interference signatures. The key idea of this work is using ZigBee interfaces to detect Wi-Fi access points which can significantly save energy compared with using Wi-Fi interfaces. Furthermore, a K-nearest neighbor method with the Manhattan distance is introduced to increase the accuracy of the localization system. The experimental results show that the proposed solution can save 68% of energy compared with the method using Wi-Fi interfaces. The accuracy is also improved by 87% compared to state-of-the-art Wi-Fi fingerprint-based approaches.

#### 2) Public Place Monitoring and Access Scheduling

In a Zigbee system, there is a central hub, known as the network coordinator, to control other connected devices in the network. Thus, Zigbee can be used to control the number of people in indoor environments. Specifically, when a person equipped with a Zigbee-enabled device (e.g., ID card or member card) enters the place, the device will connect to the Zigbee central hub. As such, the central hub is able to calculate the total number of people inside the place at a given time. Based on this information, the local manager can ask people to queue before entering the place if it is too crowded.

*Summary:* Zigbee technology can play an important role in enabling social distancing during pandemic outbreaks. However, Zigbee is a relatively new technology and has not been widely adopted in our daily life, and thus limiting its practical applications. Nevertheless, with the support from leading companies such as Amazon, Google, Apple, and Texas Instruments [160], the number of Zigbee-enable devices is expected to explosively increase in the near future. Furthermore, combining Zigbee with other technologies (e.g., Wi-Fi [163]) is also a promising research direction to improve the performance of localization systems in terms of accuracy and robustness.

### G. RFID

RFID plays a key role in real-time object localizing and tracking [150]. An RFID localization system usually consists of three main components: (i) RFID readers, (ii) RFID tags, and (iii) a data processing system [151]. Typically, RFID tags can be categorized into two types: (i) active tags and (ii) passive tags. A passive RFID tag can operate without requiring any power source, and it is powered by the electromagnetic field generated by the RFID reader. In contrast, an active RFID tag has its own power source, e.g., a battery, and continuously broadcasts its own signals. Active RFID tags are usually used in localization systems. Thus, RFID technology can be considered a potential technology to practice social distancing.

#### 1) Crowd Detection

One potential application of RFID technology is locating users in the indoor environment based on recent RFID-based localization solutions [150], [154]. To that end, each user is equipped with an RFID tag, e.g., the staff ID or member cards. Based on the backscattered signals from the RFID tag, the RFID reader can determine the location of the user. If there are too many people in the same area, the system can notify the authorities to take appropriate actions, e.g., force people to leave the area to practice social distancing. Several recent mechanisms in the literature can be adopted to make this application possible during pandemic outbreaks. In [152], the authors propose an RFID-based localization system for indoor environments with high localization granularity and accuracy. The key idea of this solution is reducing the RSSI shifts, localization error, and computational complexity by using Heron-bilateration estimation and Kalman-filter drift removal. In [153], the authors propose to use a moving robot to enhance the accuracy of a real-time RFID-based localization system. In particular, the robot is able to perform Simultaneous Localization and Mapping (SLAM), and thus it can continuously interrogate all RFID tags in its area. Then, based on passive RFID tags at known locations, we can estimate the location of target tags by properly manipulating the measured backscattered power. Alternatively, in [150], the authors propose to equipped two RFID tags at the target instead of only one as in conventional solutions to improve the accuracy of localization techniques. Adding one more RFID tag possesses several advantages: (i) easy to implement and adjust the RFID reader’s antenna, (ii) enabling fine-grained calculation, and (iii) enabling accurate calibration. The experimental results then show that equipping two tags at the user can greatly increase the localization accuracy of the system.

However, the RFID technology has several limitations due to the fact that both the receiver and the RF source are in the RFID reader. Specifically, the modulated signals backscattered from the RFID tag are strongly affected by the round-trip path loss from the receiver and the RF source. In addition, the RFID system can also be affected by the near-far problem [155]. To address these problems, a few recent works propose to use bistatic and ambient backscattered communication technologies (extended version of RFID) for localization [156], [157]. The key idea is separating the RF source from the receiver. The RF source now can be a dedicated carrier emitter or an ambient RF source. The tag can then transmit data to the receiver by backscattering the RF signals generated by the RF source. Based on the received signals, the receiver can estimate the location of the tag. In [157], the authors propose a localization system based on backscatter communications to locate patients in a hospital. In particular, each patient is equipped with a backscatter tag which can backscatter signals broadcast by an RF source. Then, the location of a patient can be detected by a localization algorithm, namely Remix, based on the backscattered signals from the backscatter tags. Remix consists of two processes. First, the algorithm approximates the distance from the tag to the receiver based on the backscattered signals. Second, the signal paths are modeled with linear splines. Then, an optimization problem is solved to find the effective distances corresponding to the paths that are close to the actual paths from the tag to the receiver. As a result, Remix can accurately estimate the position of the backscatter tag by modeling the spline structure. Based on the users’ locations, Remix can detect crowds in hospitals and advice the authorities to take appropriate actions to practice social distancing. Note that this solution can also be deployed to detect crowds in other places such as workplaces, schools, and supermarkets where backscatter tags can be easily attached to users/customers’ cards, e.g., staff cards, student cards, and member cards.

#### 2) Public Place Monitoring and Access Scheduling

Another application of RFID in social distancing is monitoring the number of people inside a place, e.g., a building or supermarket. In particular, an RFID reader will be deployed at the main gate of a place, and users are equipped with RFID tags (can be either active and passive tags). The users’ tags can broadcast their ID (active) or send their ID upon receiving RF signals from the RFID reader (passive). When a user enters the place, the RFID reader can receive the user’s ID and increase the counter value. As such, the RFID reader can calculate the number of people inside the place. If there are too many people, the system can notify the local manager to force people to queue before entering the place to practice social distancing. This solution can be deployed in supermarkets or workplaces where the customers/staff usually have member/staff ID cards which can be equipped with RFID tags.

*Summary:* RFID technology is a potential solution to enable social distancing. However, unlike other wireless technologies, RFID technology has not been widely adopted in practice due to its complexity in implementation. Specifically, to be able to detect the location of people by using RFID technology, they need to be equipped with RFID tags. However, RFID tags are not readily available likes Wi-Fi access points or Bluetooth. Thus, applications of RFID technology for social distancing are still limited in practice.

Table 2 summarizes the technologies discussed in this Section. Technologies that have a wide communication range such as cellular and GNSS are effective solutions for the scenarios where it is necessary to track people’s location over a large area (e.g., the infected movement data scenario). On the other hand, technologies with a shorter communication range (e.g., Wi-Fi, Bluetooth, Zigbee, and RFID) are more suitable for scenarios that involve indoor environments such as public place monitoring. Moreover, technologies that can achieve a high positioning accuracy (e.g., Ultra-wideband and Bluetooth) can be employed to keep a safe distance between any two people, except for GNSS since it requires a high cost to maintain sufficient accuracy. Furthermore, most of these technologies are ready to be implemented and integrated with existing systems such as smartphones. However, user privacy is an open issue for most wireless technologies. Furthermore, other emerging wireless technologies such as LoRaWAN, Z-Wave, and NFC [158] have not been well investigated in the literature for positioning systems, and thus they could be potential research directions for social distancing in the future.

**TABLE 2:** Summary of Wireless Technologies Applications to Social Distancing

Technology	Range	Accuracy	Cost	Privacy	Scalability	Indoor/Outdoor	Readily available devices
Wi-Fi [20], [51], [53], [55], [59], [168]	Typically up to 50 m indoors and 100 m outdoors [164]	1–5 m [54]	Low	Low	High	Indoor	Mobile phones, smart home, smart city [66], [168]
Cellular [86], [87], [90], [93], [97]	Short to Long	Less than 50 cm [77]	High	Low to High	High	Both	Smartphone, Smartwatch, Mobile phones [226], Intelligent Transportation Systems [213], [220], [221]
Ultra- wideband [121]–[124], [128], [129], [131], [132]	Short to Medium	Less than 10 cm [121], [122], [128]	Medium	High	Medium	Both	Smartphone [131], Smart industry [121]–[123], Public transport management [124], Commercial vehicles [121], [124]
Bluetooth [103], [105], [108], [111]–[116], [168]	Outdoor: 55–78 m. Office: 15–19 m. Home: 26–35 m [165]	1–2 m [114]	Low	Low	High	Both	Mobile phones, smart home, smart city [113], [117], [168]
Zigbee [159], [162], [163], [168]	Up to 300+ m (line of sight). Up to 100 m indoor [160]	3–5 m [159]	Low to Medium	Low	Low	Indoor	Mobile phones, smart home, smart city [171]–[173]
GNSS [136], [138]–[140], [142]–[144], [146]–[149]	World-wide coverage	Outdoor less than 10 cm [141], indoor inm	High	Medium	High	Outdoor	Mobile phones [217], [219], [224], Intelligent Transportation Systems [213], [214]
RFID [150], [152], [154], [168]	Active: 100 m or more. Passive: 10 m [166]	Less than 1 m [152]	Low to Medium	Low	Medium	Indoor	Mobile phones, smart home, smart city [169], [170]

## IV. Conclusion

Social distancing has been considered a crucial measure to prevent the spread of contagious diseases such as COVID-19. In this article, we have presented a comprehensive survey on how technologies can enable, encourage, and enforce social distancing. Firstly, we provided an overview of the social distancing, discussed its role in the current COVID-19 pandemic, and introduced various practical social distancing scenarios where the technologies can be leveraged. We then presented and reviewed various wireless technologies to encourage and facilitate social distancing measures. For each technology, we provided an overview, examined the state-of-the-art, and discussed how it can be utilized in different social distancing scenarios. Other emerging technologies, such as machine learning, computer vision, thermal, ultrasound, etc., as well as open issues in social distancing implementation and their potential solutions will be discussed in the companion paper Part II [1].
